# Supplementary material for: Efficient use of cement and concrete to reduce reliance on supply-side technologies for net-zero emissions
Source: Nat Commun. 2022 Jul 18;13:4158. doi: 10.1038/s41467-022-31806-2 (PMC9293885; doi:10.1038/s41467-022-31806-2)
Supplement: Supplementary file 1 — Supplementary Information [file 41467_2022_31806_MOESM1_ESM.pdf]

Supplementary Information

Efficient use of cement and concrete to reduce reliance on supply-side technologies for net-zero emissions

Takuma Watari [1,2], Zhi Cao [3], Sho Hata [1,4], Keisuke Nansai [1]

1 Material Cycles Division, National Institute for Environmental Studies, Japan

2 Institute for Sustainable Futures, University of Technology Sydney, Australia

3 Energy and Materials in Infrastructure and Buildings (EMIB), University of Antwerp, Belgium

4 Graduate School of Frontier Sciences, The University of Tokyo, Japan

Number of pages: 71, figures: 34, tables: 29

1. Modeling framework

1.1 Mapping the cement and concrete cycle

The system model developed in this work is centered on concrete and quantifies the flows and stocks of other materials and resources including cement, aggregates, and water.

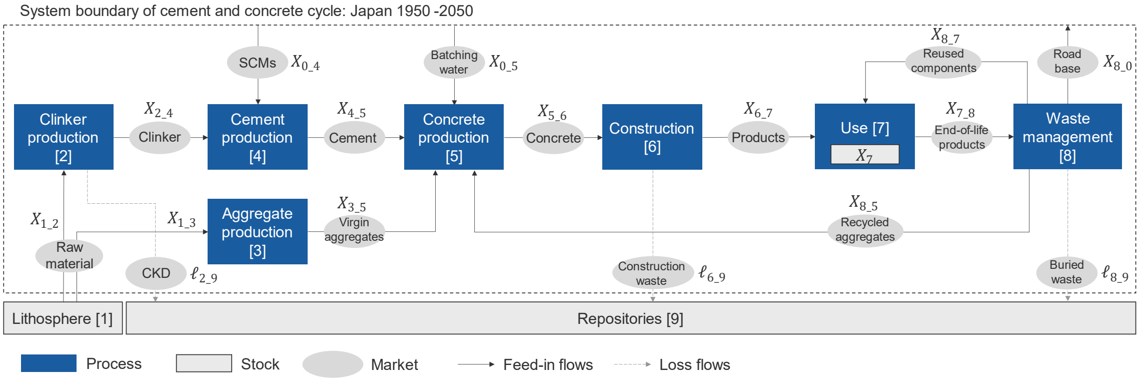

Fig. S1 System definition of cement and concrete cycle in Japan.

The concrete-related flows and stocks are represented by the following equations:

$$X_{5\_6,\beta}\ (t) = \mathrm{CA}_\beta\ (t) \times \mathrm{MI}_\beta(t) \tag{1}$$

$$X_{6\_7,\beta}\ (t) = X_{5\_6,\beta}\ (t) \times \lambda(t) \tag{2}$$

$$X_{7,\beta}(t) = \sum_{t'=0}^{t} (X_{6\_7,\beta}(t') + X_{8\_7,\beta}(t') - X_{7\_8,\beta}(t')) \tag{3}$$

$$X_{7\_8,\beta}(t) = \sum_{t'=0}^{t} ((X_{6\_7,\beta}(t') + X_{8\_7,\beta}(t')) \times \phi_\beta(t - t')) \tag{4}$$

$$\ell_{6\_9}\ (t) = \sum_{\beta} \left(X_{5\_6,\beta}\ (t) - X_{6\_7,\beta}\ (t)\right) \tag{5}$$

where the system variables and parameters are defined as follows. t: Time. β: Application category. CA: Construction activity. MI: Material intensity. X_{5_6}: Finished concrete. X_{6_7}: Inflow of concrete-containing products. ℓ_{6_9}: Construction waste. λ: Fabrication yield. X_7: In-use concrete stock. X_{8_7}: Reused components. X_{7_8}: End-of-life concrete-containing products. ϕ: Lifetime distribution.

We considered 11 application categories: residential buildings, non-residential buildings, roads, landslide and flood control, agriculture forestry and fisheries, industrial water, sewerage, harbors and airports, railways and telecommunications, parks, and waste treatment. In this case, residential and non-residential buildings are further broken down into six

categories according to their structure type: wood, steel-reinforced concrete, reinforced concrete, steel frame, concrete block, and others.

The end-of-life concrete is either reused as components (X_{8_7}), downcycled into aggregate for concrete (X_{8_5}) and road base material (X_{8_0}), or landfilled (ℓ_{8_9}).

$$X_{8\_7}(t) = \delta(t) \times \sum_{\beta} X_{7\_8,\beta}(t) \tag{6}$$

$$X_{8\_5}(t) = \gamma(t) \times \sum_{\beta} X_{7\_8,\beta}(t) \tag{7}$$

$$X_{8\_0}(t) = \pi(t) \times \sum_{\beta} X_{7\_8,\beta}(t) \tag{8}$$

$$\ell_{8\_9}\ (t) = (1 - \delta(t) - \gamma(t) - \pi(t)) \times \sum_{\beta} X_{7\_8,\beta}(t) \tag{9}$$

where δ, γ, and π denote the reuse, downcycling for concrete aggregate, and downcycling for road base rates, respectively. In this case, ℓ_{8_9} includes so-called hibernation or missing stock, which remains after it is no longer used or is left mixed with soil and sand at the site without being removed from the demolition site [1,2].

Concrete produced to meet the domestic demand for building and infrastructure manufacturing mainly consists of cement (X_{4_5}), virgin aggregates (X_{3_5}), recycled aggregates (X_{8_5}), and batching water (X_{0_5}).

$$\sum_{\beta} X_{5\_6,\beta}\ (t) = X_{4\_5}(t) + X_{3\_5}(t) + X_{8\_5}(t) + X_{0\_5}(t) \tag{10}$$

In this case, the cement consists of clinker (X_{2_4}) or supplementary cementitious materials (X_{0_4}) such as blast furnace slag or fly ash.

$$X_{4\_5}(t) = X_{2\_4}(t) + X_{0\_4}(t) \tag{11}$$

$$X_{2\_4}(t) = X_{1\_2}(t) \times (1 - \mathrm{CKD}) \tag{12}$$

$$\ell_{2\_9}\ (t) = X_{2\_4}(t) - X_{1\_2}(t) \tag{13}$$

where X_{1_2} represents the raw material, and CKD expresses the multiplication of the cement kiln dust generation rate and its landfill rate. Virgin aggregates are supplied from several sources: crushed stone, sea sand, land sand, mountain sand, river sand, and others including artificial lightweight aggregate.

1.2 Calculating CO_2 emissions

The CO_2 emissions associated with the cement and concrete cycle are calculated based on each process's detailed energy consumption and emission factor dataset. This approach allows us to track the CO_2 emissions associated with electrical and thermal energy consumption, and the CO_2 emissions associated with chemical reactions (the conversion of $CaCO_3 \rightarrow CaO + CO_2$ in the kiln), over the entire concrete cycle. The emission sources considered in this work are primarily divided into six major categories: cement production, virgin aggregate production, recycled aggregate production, concrete mixing, concrete on-site placement, and transportation activities. Emissions from the use and dismantling phases are excluded in the model, as it is difficult to assign them to a single material.

1.3 Calculating CO_2 uptake

The CO_2 uptake from concrete carbonation is estimated using a physicochemical model [3–5]. The model estimates the total CO_2 uptake over the lifecycle (U_{total}) considering four sinks: concrete (U_{con}), mortar (U_{mor}), construction waste (U_{waste}), and cement kiln dust (U_{CKD}).

$$U_{\mathrm{total}} = \sum U_{\mathrm{con}} + \sum U_{\mathrm{mor}} + \sum U_{\mathrm{waste}} + U_{\mathrm{CKD}} \quad (14)$$

Uptake by concrete

The CO_2 uptake by concrete is calculated by summing the uptakes during the use stage ($U_{\mathrm{con,use}}$), demolition stage ($U_{\mathrm{con,dem}}$), and secondary use stage ($U_{\mathrm{con,sec}}$).

$$\sum U_{\mathrm{con}} = U_{\mathrm{con,use}} + U_{\mathrm{con,dem}} + U_{\mathrm{con,sec}} \quad (15)$$

In this case, uptake by concrete during the use stage ($U_{\mathrm{con,use}}$) is calculated by the following equations:

$$U_{\mathrm{con,use}} = W_{\mathrm{con,use}} \times C_{\mathrm{clinker}} \times f_{\mathrm{CaO}} \times \gamma \times M_r \quad (16)$$

$$\mathrm{W}_{\mathrm{con,use}} = \sum_{i=1}^{n} V_{\mathrm{con},i} \times C_{\mathrm{con},i} = \sum_{i=1}^{n} d_{\mathrm{con},i} \times \frac{W_{\mathrm{con},i}}{T_w} \quad (17)$$

where the variables are defined as follows. $W_{\mathrm{con,use}}$: Mass of carbonated cement used in concrete. C_{clinker}: Clinker-to-cement ratio. f_{CaO}: Average mass content of CaO in clinker. γ: Proportion of CaO within fully carbonated cement that converts to $CaCO_3$ for concrete cement. M_r: Mole ratio of CO_2 to CaO. $V_{\mathrm{con},i}$: Volume of carbonated concrete in concrete class i. $C_{\mathrm{con},i}$: Concrete cement content in concrete class i. $d_{\mathrm{con},i}$: Carbonated depth of concrete in concrete class i. $W_{\mathrm{con},i}$: Mass of cement used in concrete class i. T_w: Average thickness of concrete structures.

The carbonated depth of concrete in concrete class i ($d_{\text{con},i}$) over a certain period of time (t_{use}) is calculated based on Fick's diffusion law.

$$d_{\text{con},i} = k_{\text{con},i} \times \sqrt{t_{\text{use}}} \tag{18}$$

where $k_{\text{con},i}$ is the concrete carbonation rate coefficient in concrete class i, which is adjusted to account for the effects of exposure conditions ($\beta_{i,\text{ec}}$), cement additives (β_{ad}), CO_2 concentration (β_{CO_2}), and coating and cover (β_{cc}):

$$k_{\text{con},i} = \beta_{i,\text{ec}} \times \beta_{\text{ad}} \times \beta_{\text{CO}_2} \times \beta_{\text{cc}} \tag{19}$$

Similarly, the CO_2 uptake by concrete during the demolition stage ($U_{\text{con,dem}}$) is calculated based on the mass of carbonated cement used in concrete during the demolition stage ($W_{\text{con,dem}}$).

$$U_{\text{con,dem}} = W_{\text{con,dem}} \times C_{\text{clinker}} \times f_{\text{CaO}} \times \gamma \times M_r \tag{20}$$

$$W_{\text{con,dem}} = \sum_{i=1}^{n}((W_{\text{con},i} - W_{\text{con,use},i}) \times \ F_{\text{dem},i}) \tag{21}$$

where $F_{\text{dem},i}$ is the carbonated fraction of demolished concrete in concrete class i during the demolition stage. The concrete carbonation at the demolition stage is modeled by assuming that the particles of demolition waste are spherical, since concrete structures are usually crushed into small pieces in order to recycle steel and facilitate subsequent transport of demolition waste. Thus, the carbonated fraction of demolished concrete is estimated as follows:

$$F_{\text{dem},i} = \begin{cases} 100\% - \dfrac{\int_a^b \frac{4}{3}\pi(R - d_{\text{con,dem},i})^3}{\int_a^b \frac{4}{3}\pi R^3} \times 100\%, & (a \geq 2d_{\text{con,dem},i}) \\ 100\% - \dfrac{\int_{2d_{\text{con,dem},i}}^b \frac{4}{3}\pi(R - d_{\text{con,dem},i})^3}{\int_a^b \frac{4}{3}\pi R^3} \times 100\%, & (a < 2d_{\text{con,dem},i} < b) \\ 100\%, & (b \leq 2d_{\text{con,dem},i}) \end{cases} \tag{22}$$

Here, R, a, and b represent the ranges of particle size, minimum diameter, and maximum diameter, respectively. $d_{\text{con,dem},i}$ is the carbonated depth during the demolition stage and is calculated by the following equation:

$$d_{\text{con,dem},i} = k_{\text{con,dem},i} \times \sqrt{t_{\text{dem}}} \tag{23}$$

where $k_{\text{con,dem},i}$ is the carbonation rate coefficient in open air exposure conditions and t_{dem} is the average exposure time during the demolition stage.

Finally, the CO_2 uptake by concrete during the secondary use stage ($U_{\text{con,sec}}$) is calculated by the following equations:

$$U_{\text{con,sec}} = W_{\text{con,sec}} \times C_{\text{clinker}} \times f_{\text{CaO}} \times \gamma \times M_r \tag{24}$$

$$W_{\text{con,sec}} = \sum_{i=1}^{n}((W_{\text{con},i} - W_{\text{con,use},i} - W_{\text{con,dem},i}) \times F_{\text{sec},i}) \tag{25}$$

where $W_{\text{con,sec}}$ is the mass of carbonated cement used in concrete during the secondary use stage and $F_{\text{sec},i}$ is the carbonated fraction of demolished concrete during the secondary stage.

Similar to the demolition stage, the carbonated fraction of demolished concrete during the secondary stage ($F_{\text{sec},i}$) over a certain period of time during the secondary use stage (t_{sec}) is based on particle size (R), minimum diameter (a), maximum diameter (b), carbonated depths during the demolition stage ($d_{\text{con,dem},i}$), carbonated depths during the secondary use stage ($d_{\text{con,sec},i}$), total carbonated depths during demolition and secondary use stages ($d_{\text{con,total},i}$), and carbonation coefficient in buried conditions ($k_{\text{con,sec},i}$).

$$F_{\text{sec},i} = \begin{cases} 100\% - \dfrac{\int_a^b \frac{4}{3}\pi(R - d_{\text{con,sec},i})^3}{\int_a^b \frac{4}{3}\pi R^3 \times 100\%} \times 100\% - F_{\text{dem},i} & (a \geq 2d_{\text{con,total},i}) \\ 100\% - \dfrac{\int_{2d_{\text{con,dem},i}}^b \frac{4}{3}\pi(R - d_{\text{con,sec},i})^3}{\int_a^b \frac{4}{3}\pi R^3} - F_{\text{dem},i} & (a < 2d_{\text{con,total},i} < b) \\ 100\%, -F_{\text{dec},i} & (b \leq 2d_{\text{con,totoal},i}) \end{cases} \tag{26}$$

$$d_{\text{con,sec},i} = k_{\text{con,sec},i} \times (\sqrt{t_{\text{dem}} + t_{\text{sec}}} - \sqrt{t_{\text{dem}}}) \tag{27}$$

$$d_{\text{con,total},i} = d_{\text{con,dem},i} + d_{\text{con,sec},i} \tag{28}$$

Uptake by mortar

The CO_2 uptake by mortar is calculated by summing the uptakes by mortar used for rendering and plastering ($U_{\text{mor,rp}}$), masonry ($U_{\text{mor,ma}}$), repairing and maintenance ($U_{\text{mor,rm}}$), during demolition stage ($U_{\text{mor,dem}}$), and during secondary use stage ($U_{\text{mor,sec}}$).

$$\sum U_{\text{mor}} = U_{\text{mor,rp}} + U_{\text{mor,ma}} + U_{\text{mor,rm}} + U_{\text{mor,dem}} + U_{\text{mor,sec}} \tag{29}$$

In this case, the uptake by mortar during the demolition and secondary use stage is modeled using the same particle size assumptions and modeling procedures as for the concrete. We next describe the modeling procedure for the use stage.

The CO_2 uptake by mortar used for rendering and plastering ($U_{\text{mor,rp}}$) is calculated based on the mass of cement used in the mortar (W_{mor}), the percentage of mortar used for rendering and plastering (r_{rp}), the carbonated fraction of cement used for rendering and plastering (F_{rp}), the clinker-to-cement ratio (C_{clinker}), the average mass content of CaO in the

clinker (f_{CaO}), the proportion of CaO within fully carbonated cement that converts to $CaCO_3$ for mortar cement (γ_1), and the mole ratio of CO_2 to CaO (M_r).

$$U_{\mathrm{mor,rp}} = W_{\mathrm{mor}} \times r_{\mathrm{rp}} \times F_{\mathrm{rp}} \times C_{\mathrm{clinker}} \times f_{\mathrm{CaO}} \times \gamma_1 \times M_r \tag{30}$$

Here, the carbonated fraction of cement used for rendering and plastering (F_{rp}) is calculated based on the depth of the carbonated mortar used for rendering and plastering (d_{rp}) and the thickness of the rendering and plastering (T_{rp}).

$$F_{\mathrm{rp}} = \frac{d_{\mathrm{rp}}}{T_{\mathrm{rp}}} \times 100\% \tag{31}$$

where the depth of the carbonated mortar used for rendering and plastering (d_{rp}) over a certain period of time (t_{use}) is calculated based on the carbonation rate of mortar (k_{mor}).

$$d_{\mathrm{rp}} = k_{\mathrm{mor}} \times \sqrt{t_{\mathrm{use}}} \tag{32}$$

The CO_2 uptake by mortar used for masonry ($U_{\mathrm{mor,ma}}$) is estimated as the sum of the uptakes by masonry rendered on both sides ($U_{\mathrm{mor,ma,both}}$), rendered on only one side ($U_{\mathrm{mor,ma,one}}$), and without rendering ($U_{\mathrm{mor,ma,non}}$).

$$U_{\mathrm{mor,ma}} = U_{\mathrm{mor,ma,both}} + U_{\mathrm{mor,ma,one}} + U_{\mathrm{mor,ma,non}} \tag{33}$$

in which

$$U_{\mathrm{mor,ma,both}} = W_{\mathrm{mor}} \times r_{\mathrm{ma}} \times r_{\mathrm{both}} \times F_{\mathrm{ma,both}} \times C_{\mathrm{clinker}} \times f_{\mathrm{CaO}} \times \gamma_1 \times M_r \tag{34}$$

$$U_{\mathrm{mor,ma,one}} = W_{\mathrm{mor}} \times r_{\mathrm{ma}} \times r_{\mathrm{one}} \times F_{\mathrm{ma,one}} \times C_{\mathrm{clinker}} \times f_{\mathrm{CaO}} \times \gamma_1 \times M_r \tag{35}$$

$$U_{\mathrm{mor,ma,non}} = W_{\mathrm{mor}} \times r_{\mathrm{ma}} \times r_{\mathrm{non}} \times F_{\mathrm{ma,non}} \times C_{\mathrm{clinker}} \times f_{\mathrm{CaO}} \times \gamma_1 \times M_r \tag{36}$$

where r_{ma} is the percentage of mortar used for masonry. r_{both}, r_{one}, and r_{non} represent the percentages of masonry walls with rendering on both sides, with rendering on only one side, and without rendering, respectively. $F_{\mathrm{ma,both}}$, $F_{\mathrm{ma,one}}$, and $F_{\mathrm{ma,non}}$ are the carbonated fractions of cement used for masonry rendered on both sides, rendered on only one side, and without rendering, respectively. These variables are calculated as follows:

$$F_{\mathrm{ma,both}} = \frac{d_{\mathrm{ma,both}}}{T_w} \times 100\% \tag{37}$$

$$F_{\mathrm{ma,one}} = \frac{d_{\mathrm{ma,one}}}{T_w} \times 100\% \tag{38}$$

$$F_{\mathrm{ma,non}} = \frac{d_{\mathrm{ma,non}}}{T_w} \times 100\% \tag{39}$$

where $d_{\mathrm{ma,both}}$, $d_{\mathrm{ma,one}}$, and $d_{\mathrm{ma,non}}$ are the depths of carbonated mortar for masonry rendered on both sides, rendered on only one side, and without rendering, respectively. T_w is the wall thickness.

Here, the depth of carbonated mortar for masonry over a certain period of time (t_{use}) is calculated by the following equations:

$$d_{\text{ma,both}} = \begin{cases} 0, & (t_{\text{use}} \le t_r) \\ 2(k_{\text{mor}} \times \sqrt{t_{\text{use}}} - T_{\text{rp}}), & (t_{\text{use}} > t_r) \end{cases} \quad (40)$$

$$d_{\text{ma,one}} = \begin{cases} k_{\text{mor}} \times \sqrt{t_{\text{use}}}, & (t_{\text{use}} \le t_r) \\ k_{\text{mor}} \times \sqrt{t_{\text{use}}} + (k_{\text{mor}} \times \sqrt{t_{\text{use}}} - T_{\text{rp}}), & (t_{\text{use}} > t_r) \end{cases} \quad (41)$$

$$d_{\text{ma,non}} = 2k_{\text{mor}} \times \sqrt{t_{\text{use}}} \quad (42)$$

where t_r is the time that the mortar for rendering is fully carbonated and T_{rp} is the thickness of rendering and plastering.

Finally, the CO_2 uptake by mortar used for repairing and maintenance ($U_{\text{mor,rm}}$) is calculated as follows:

$$U_{\text{mor,rm}} = W_{\text{mor}} \times r_{\text{rm}} \times F_{\text{rm}} \times C_{\text{clinker}} \times f_{\text{CaO}} \times \gamma_1 \times M_r \quad (43)$$

$$F_{\text{rm}} = \frac{d_{\text{rm}}}{T_{\text{rm}}} \times 100\% \quad (44)$$

$$d_{\text{rm}} = k_{\text{mor}} \times \sqrt{t_{\text{use}}} \quad (45)$$

where r_{rm} is the percentage of mortar used for repairing and maintenance, F_{rm} is the carbonated fraction of cement used for repairing and maintenance, d_{rm} is the depth of carbonated mortar used for repairing and maintaining, and T_{rm} is the thickness of repairing and maintenance.

Uptake by construction waste

The CO_2 uptake by construction waste (U_{waste}) is estimated as the sum of the uptakes by concrete waste ($U_{\text{con,waste}}$) and mortar waste ($U_{\text{mor,waste}}$).

$$\sum U_{\text{waste}} = U_{\text{con,waste}} + U_{\text{mor,waste}} \quad (46)$$

The CO_2 uptake by construction concrete waste ($U_{\text{con,waste}}$) and mortar waste ($U_{\text{mor,waste}}$) is calculated based on the mass of cement used for concrete (W_{con}), the mass of cement used for mortar (W_{mor}), the loss rate of cement in the construction stage (l), the carbonated mass fraction of construction concrete waste (F_{con}), the carbonated mass fraction of construction mortar waste (F_{mor}), the clinker-to-cement ratio (C_{clinker}), the average mass content of CaO in clinker (f_{CaO}), the proportion of CaO within fully carbonated cement that converts to $CaCO_3$ for concrete cement (γ), and the mole ratio of CO_2 to CaO (M_r).

$$U_{\text{con, waste}} = W_{\text{con}} \times l \times F_{\text{con}} \times C_{\text{clinker}} \times f_{\text{CaO}} \times \gamma \times M_r \tag{47}$$

$$U_{\text{mor,waste}} = W_{\text{mor}} \times l \times F_{\text{mor}} \times C_{\text{clinker}} \times f_{\text{CaO}} \times \gamma_1 \times M_r \tag{48}$$

Given the fineness of construction waste, concrete waste is assumed to be completely carbonated over five years ($F_{\text{con}} = 0.2$), whereas mortar waste is assumed to be completely carbonated in one year ($F_{\text{mor}} = 1$).

Uptake by cement kiln dust

The CO_2 uptake by cement kiln dust (CKD) is calculated based on the mass of cement production (W_{pro}), the clinker-to-cement ratio (C_{clinker}), the CKD generation rate based on clinker (r_{CKD}), the proportion of CKD landfilled (r_{landfill}), the average CaO content of CKD ($f_{\text{CaO,CKD}}$), the proportion of CaO within fully carbonated CKD that converts to $CaCO_3$ (γ_2), and the mole ratio of CO_2 to CaO (M_r).

$$U_{\text{CKD}} = W_{\text{pro}} \times C_{\text{clinker}} \times r_{\text{CKD}} \times r_{\text{landfill}} \times f_{\text{CaO,CKD}} \times \gamma_2 \times M_r \tag{49}$$

where $f_{\text{CaO,CKD}}$ is assumed to be 100%.

1.4 Linking material flows with final demand drivers

To better understand the demand-side drivers of the current cement and concrete cycle, we link estimated material flow data to final demand drivers based on the input-output approach. This study employs a fixed-capital matrix-augmentation method that allows us to define the number of sectors in the fixed-capital matrix arbitrarily according to data availability and to obtain explicitly the contribution of fixed-capital formation to the capital-embodied material footprint [6]. A capital-embodied material footprint for final demand can be formulated as follows:

$$\mathbf{P} = \mathbf{W}(\mathbf{I} - \mathbf{A}')^{-1}\mathbf{y} = \mathbf{W}\mathbf{L}'\mathbf{y} \tag{50}$$

$$\mathbf{A}' = \begin{pmatrix} \mathbf{A} & \mathbf{B} \\ \mathbf{C} & \mathbf{0} \end{pmatrix} \tag{51}$$

where, $\mathbf{W} = (w_{kj})$ is a matrix, with elements w_{kj} representing the direct input of material *k* per unit production in sector *j*, and vector $\mathbf{y} = (y_i)$ represents the final demand for commodity *i*. Matrix $\mathbf{P} = (p_{kj})$ represents the capital-embodied material (*k*) footprint associated with final demand, which includes material use through the industrial process as well as fixed-capital formation. Matrix $\mathbf{A} = (a_{ij})$ consists of element a_{ij}, which represents the inputs of commodity *i* to the unit production activity in sector *j*. Matrix $\mathbf{L}'$ is the Leontief inverse matrix representing the spillover effect of the supply chain including fixed-capital. Matrix $\mathbf{B} = (b_{il})$, the capital formation matrix, represents the inputs of commodity *i* to *l* types of fixed-capital formation sector. Matrix $\mathbf{C} = (c_{lj})$, the capital utilization matrix, describes *l* types of fixed-capital utilization with respect to unit production in sector *j*.

When matrix $\mathbf{A}$ and vector $\mathbf{y}$ include imported materials, matrix P represents the total material footprint of domestic and imported products. In this study, we focus on domestic production only; the flows of imported materials are excluded from matrix $\mathbf{A}$ and vector $\mathbf{y}$ by defining the matrix as $\mathbf{A}^{d\prime} = (\mathbf{I} - \widehat{\mathbf{m}})\mathbf{A}'$ and the vector as $\mathbf{y}^d = (\mathbf{I} - \widehat{\mathbf{m}})\mathbf{y}$. Thus, equation (50) is formulated as $\mathbf{P} = \mathbf{W}(\mathbf{I} - \mathbf{A}^{d\prime})\mathbf{y}^d = \mathbf{W}\mathbf{L}^{d\prime}\mathbf{y}^d$. Here, each element m_i of the vector $\mathbf{m} = (m_i)$ is the import ratio of commodity *i*.

Matrix $\mathbf{A}$ is determined using the 2015 Japanese Input-Output Table (JIOT), which consists of 390 production sectors (*i* = 1…390, *j* = 1…390). Matrix $\mathbf{B}$ is obtained from the fixed-capital formation table, which consists of 116 fixed-capital formation sectors (*l* = 1…116), in the supplementary material of the JIOT. Since the JIOT does not provide matrix $\mathbf{C}$, this study constructs the matrix by establishing which capital formation is required for which production sectors. The magnitude of the capital formation requirements is estimated under the assumption that the depreciation of fixed-capital for commodity production in a given year is equal to the amount of new fixed-capital invested in that year [6].

For matrix $\mathbf{W}$, we link the application categories of cement flows in 2019 to the industrial sectors of the JIOT. Eleven cement flow categories for each application (k = 1 ... 11) are defined in $\mathbf{W}$, and the sector in JIOT corresponding to each application is defined. In cases where more than one sector is allocated to an application category, the allocation is made according to the annual total output of the sector, which is available from the JIOT.

2. Data

Table S1 Summary of data source.

Data	Period	Ref.
Cement supply and demand	1950-2020	[7]
Ready-mixed concrete supply and demand	2000-2020	[8]
Aggregate supply and demand	1967-2020	[9]
Newly constructed buildings	1950-2020	[10]
Newly constructed infrastructure	1950-2020	[10,11]
Material intensity of newly constructed buildings	1991-2019	[12]
Material intensity of newly constructed infrastructure	2000-2020	[7–9,11]
Average lifetime of buildings	1987-2010	[13,14]
Average lifetime of infrastructure	Static	[2,11,15,16]
End-of-life management of demolished concrete	Static	[9,17]
Cement kiln dust generation rate and landfill rate	Static	[4,18]
Fabrication yield	Static	[4]
Concrete mixtures	Static	[19]
Mortar mixtures	Static	[20]
Clinker-to-cement ratio	1992-2020	[7]
Thermal energy efficiency	2006-2019	[21]
Electrical energy efficiency	1970-2019	[21]
Fuel mix	1999-2020	[7]
Electricity mix	1970-2020	[22]
CO_2 emission factor of each fuel combustion	Static	[18]
CO_2 emission factor of each electricity generation	Static	[23]
CO_2 emission factor of aggregate production	Static	[24–27]
CO_2 emission factor of concrete mixing and placing	Static	[24–27]
CO_2 emission factor of transportation activities	Static	[24–27]

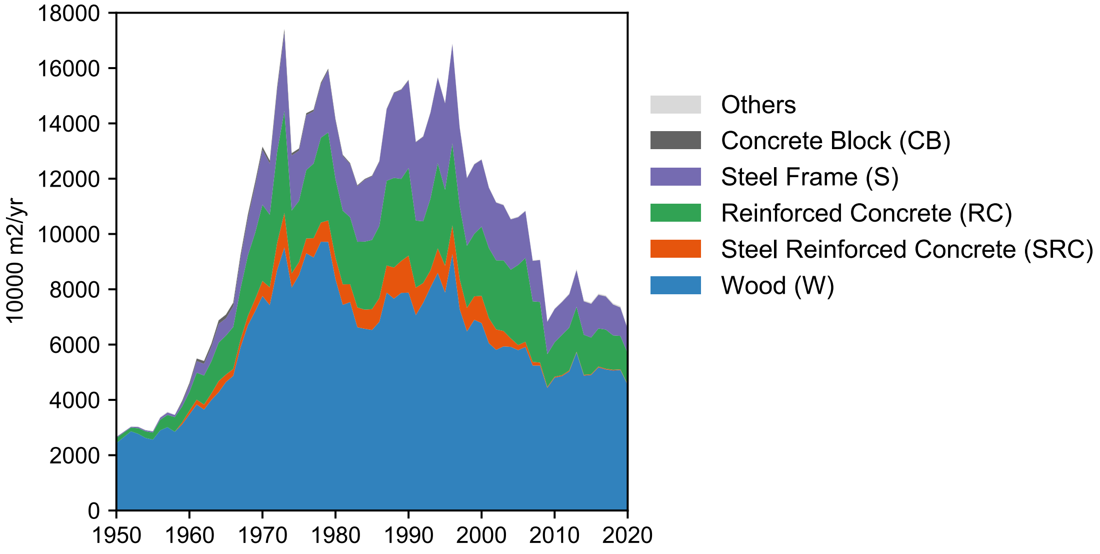

Fig. S2 Newly constructed floor area of residential buildings, 1950-2020. Data adapted from [10].

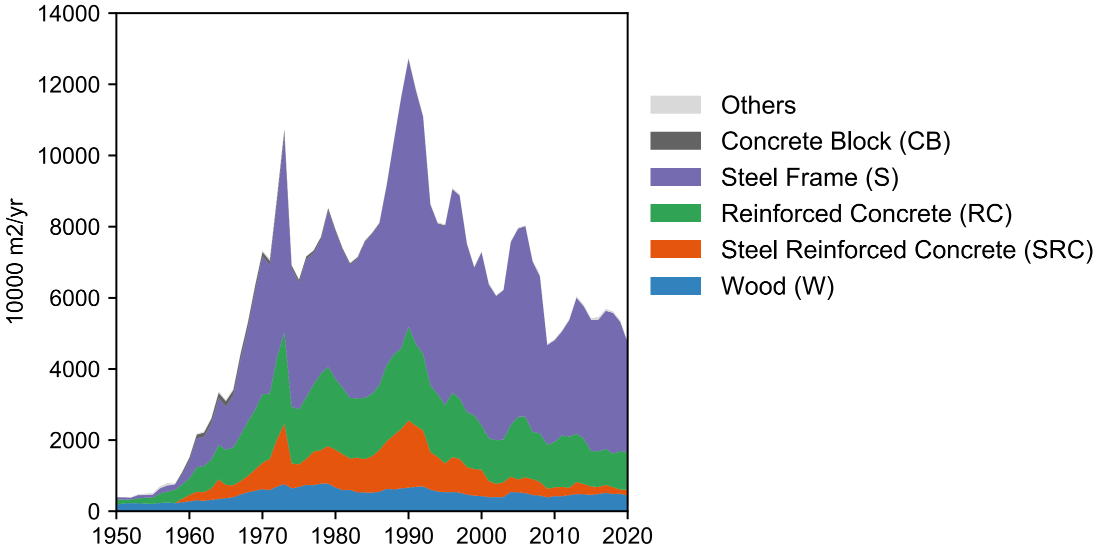

Fig. S3 Newly constructed floor area of non-residential buildings, 1950-2020. Data adapted from [10].

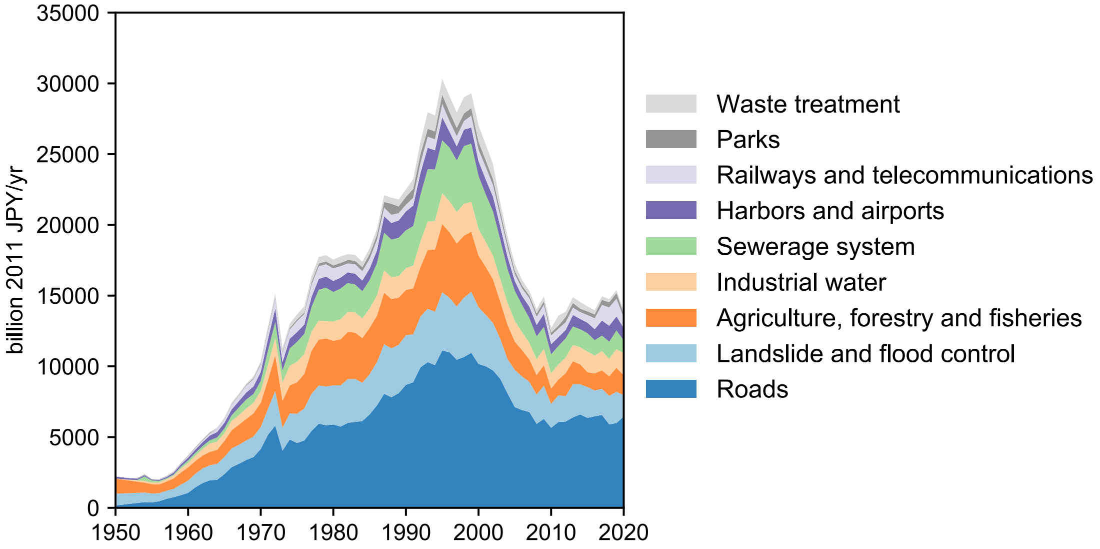

Fig. S4 Investment in newly constructed infrastructure, 1950-2020. Data adapted from [10,11].

Table S2 Concrete intensity of newly constructed residential and non-residential buildings [Unit: kg-concrete/m^2]. Data adapted from [12].

Structure type	1991	1994	1997	2000	2003	2006
Wood	514	593	600	564	543	586
Steel-reinforced concrete	2,127	2,076	1,990	2,062	2,076	1,997
Reinforced concrete	2,149	2,055	2,163	2,127	2,170	2,076
Steel frame	962	1,027	1,049	1,006	984	1,020
Concrete block	1,382	1,859	890	1,035	1,172	1,085
Others	1,427	1,522	1,338	1,359	1,389	1,353
Structure type	2009	2011	2013	2015	2017	2019
Wood	608	745	600	680	564	567
Steel-reinforced concrete	2,004	2,228	2,214	1,722	1,859	1,607
Reinforced concrete	2,554	2,279	2,243	2,243	2,207	2,263
Steel frame	1,230	1,570	1,064	1,187	1,165	1,181
Concrete block	1,599	1,599	1,599	1,599	1,599	1,599
Others	1,599	1,684	1,544	1,486	1,479	1,443

Table S3 Concrete intensity of newly constructed infrastructure [Unit: kg/thousand 2011 JPY]. Data estimated from [7–9,11].

Year	Railways and telecommunications	Harbors and airports	Roads	Other infrastructure
2000	19.9	23.6	6.6	11.3
2001	16.7	22.4	6.3	11.6
2002	17.3	20.2	6.3	11.4
2003	14.9	22.8	5.8	12.9
2004	15.2	20.9	6.1	13.3
2005	25.0	26.4	6.9	14.7
2006	21.9	25.3	6.9	15.7
2007	19.5	21.0	6.5	16.8
2008	16.6	19.9	6.8	17.1
2009	13.7	16.3	6.1	14.9
2010	18.1	14.7	6.1	17.2
2011	19.2	15.8	5.4	15.4
2012	17.7	23.8	5.1	16.2
2013	13.6	21.1	5.2	16.1
2014	14.3	18.7	5.0	16.1
2015	12.7	17.6	4.9	15.4
2016	9.8	14.2	4.9	16.6
2017	9.2	10.5	4.3	15.2
2018	8.4	9.8	4.9	15.1
2019	8.4	10.8	4.5	13.2
2020	13.1	11.7	4.1	15.1

Table S4 Average lifetime of residential buildings [Unit: year]. Numbers in parentheses indicate standard deviation. Data adapted from [13,14].

Structure type	Distribution type	1987	1997	2005	2010
Wood	Normal	42 (24)	46 (27)	56 (33)	62 (36)
Steel-reinforced concrete		54 (21)	43 (17)	45 (18)	47 (19)
Reinforced concrete		54 (21)	43 (17)	45 (18)	47 (19)
Steel frame		32 (12)	41 (15)	46 (16)	49 (18)
Concrete block		54 (21)	43 (17)	45 (18)	47 (19)
Others		32 (12)	41 (15)	46 (16)	49 (18)

Table S5 Average lifetime of non-residential buildings [Unit: year]. Numbers in parentheses indicate standard deviation. Data adapted from [13,14].

Structure type	Distribution type	1987	1997	2005	2010
Wood	Normal	42 (24)	46 (27)	56 (33)	62 (36)
Steel-reinforced concrete		41 (16)	47 (18)	51 (19)	53 (20)
Reinforced concrete		41 (16)	47 (18)	51 (19)	53 (20)
Steel frame		32 (14)	41 (15)	46 (19)	49 (22)
Concrete block		41 (16)	47 (18)	51 (19)	53 (20)
Others		32 (12)	41 (13)	46 (17)	49 (19)

Table S6 Average lifetime of infrastructure [Unit: year]. Numbers in parentheses indicate standard deviation. Data adapted from [2,11,15,16].

End-uses	Distribution type	Mean (standard deviation)
Roads	Normal	30 (15)
Landslide and flood control		50 (25)
Agriculture, forestry and fisheries		50 (25)
Industrial water		36 (18)
Sewerage		50 (25)
Harbors and airports		47 (24)
Railways and telecommunications		35 (18)
Parks		35 (18)
Waste treatment		50 (25)

Table S7 Fate of end-of-life concrete [Unit: %]. Data estimated from [9,17] and the model developed in this work.

Fate	Value
Aggregate for concrete	0.4
Road base	12.8
Landfill and hibernating stock*	86.9
Asphalt concrete	0.0

* Since the actual situation of hibernating stocks has not yet been well reported and understood, we simply assume that 30% of this category is landfill and 70% is hibernating stock, based on the literature [2,28].

Table S8 Concrete mixtures [Unit: kg/kg-concrete]. Data adapted from [19].

Type	Value
Cement	0.126
Aggregates	0.800
Water	0.075

Table S9 Mortar mixtures [Unit: kg/kg-mortar]. Data adapted from [20].

Type	Value
Cement	0.233
Aggregates	0.651
Water	0.116

Table S10 Aggregate sources [Unit: %]. Data adapted from [9].

Type	Value
Crushed stone	63
Sea sand	6
Land sand	13
Mountain sand	9
River sand	3
Others	5

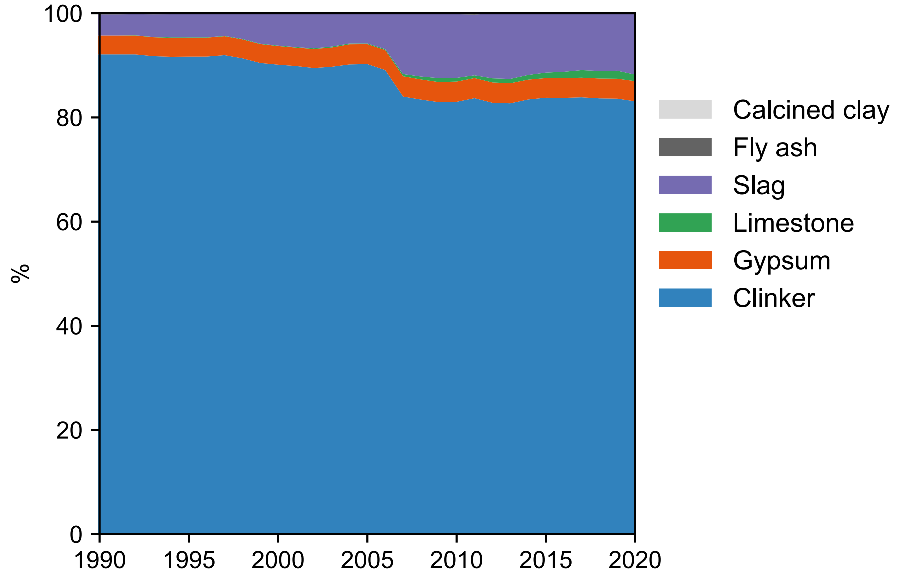

Fig. S5 Cement ingredients, 1990-2020. Data adapted from [7].

Table S11 Other system variables and parameters.

Item	Value	Unit	Ref.
Cement content of total concrete and mortar	0.138	kg-cement/kg-concrete	[7,9]
Yield of construction	1.5	%	[4,29,30]
CKD generation rate	6.0	%	[4]
Proportion of landfilled CKD	20.0	%	[18]

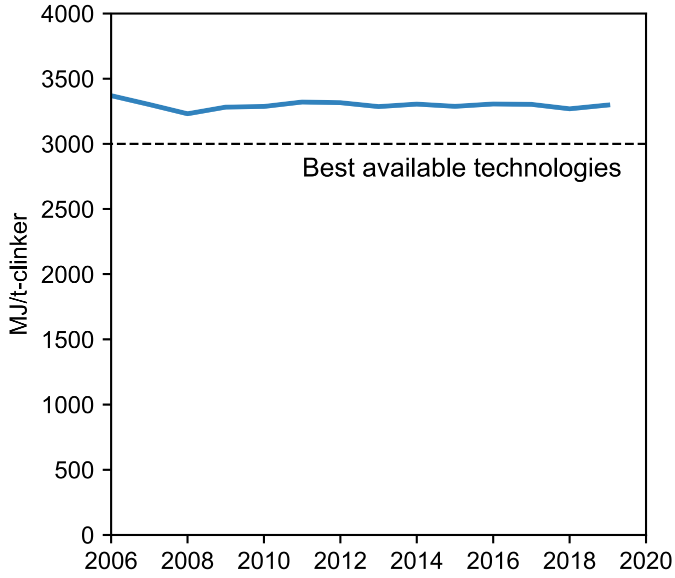

Fig. S6 Thermal efficiency in the cement kiln, 2006-2019. Data adapted from [21]. The line of best available technologies is based on [24].

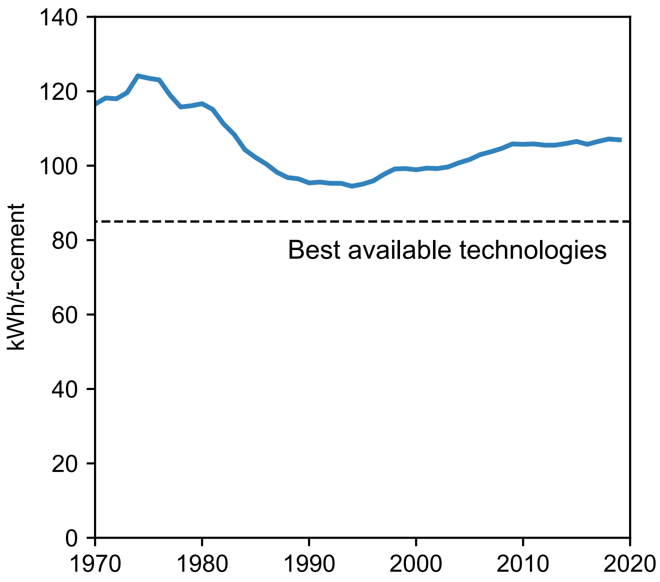

Fig. S7 Milling/grinding electrical efficiency, 1970-2019. Data adapted from [21]. The line of best available technologies is based on [24].

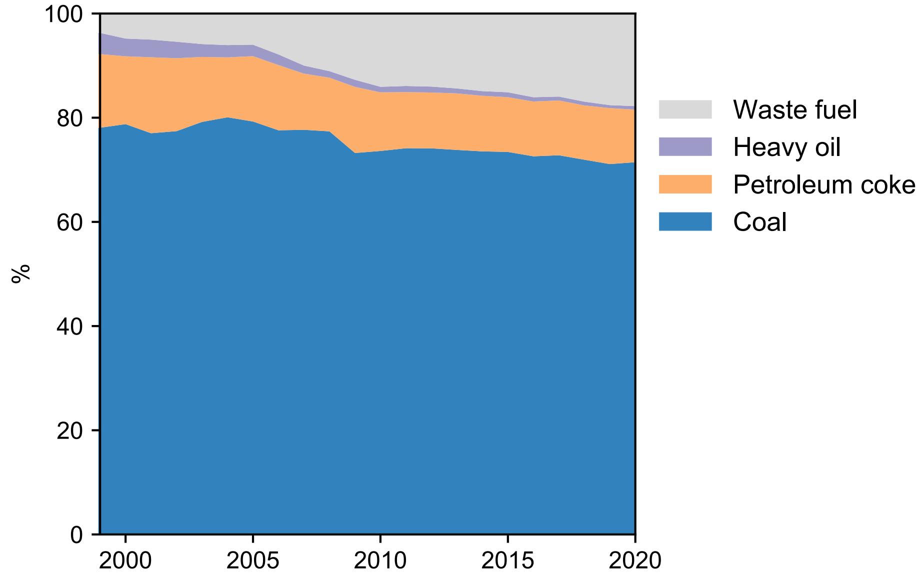

Fig. S8 Fuel mix of thermal energy consumption, 1999-2020. Data adapted from [7].

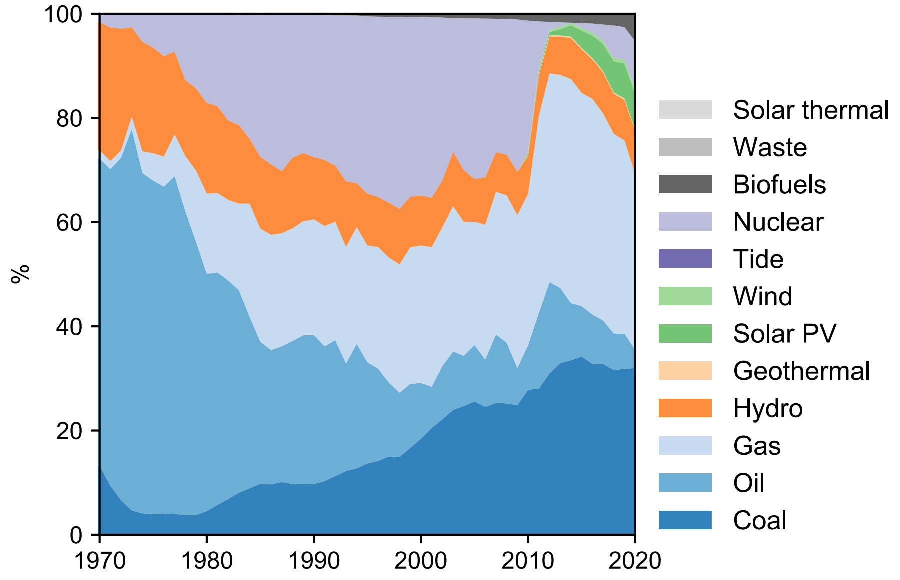

Fig. S9 Electricity generation mix, 1970-2020. Data adapted from [22].

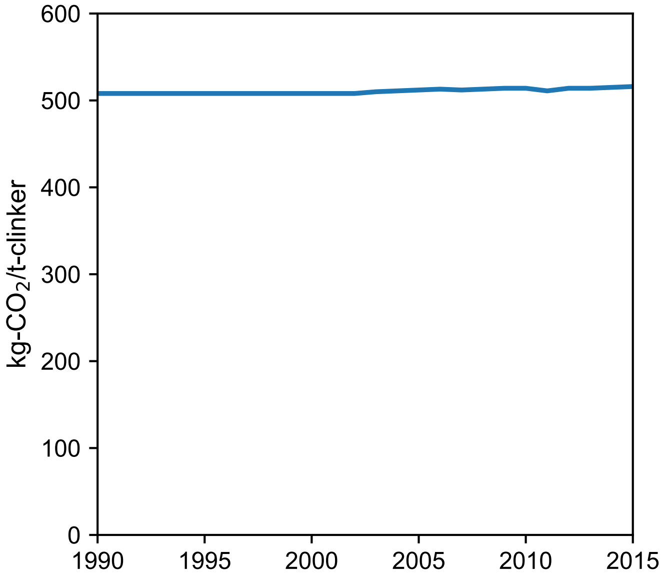

Fig. S10 Carbonate decomposition, 1990-2015. Data adapted from [18].

Table S12 CO_2 emission factor of fuel combustion [Unit: t-CO_2/MJ]. Data adapted from [18].

Type	CO_2 emission factor
Coal	90.6
Petroleum coke	93.2
Heavy oil	69.4
Natural gas	49.5
Waste fuel	69.1

Table S13 CO_2 emission factor of electricity generation [Unit: t-CO_2/GWh]. Data adapted from [23].

Type	CO_2 emission factor
Coal	908.2
Oil	657.0
Natural gas	412.9
Waste	16.8
Nuclear and renewables	0.0

Table S14 CO_2 emission factors for virgin aggregate production, recycled aggregate production, concrete mixing, concrete on-site placement, and transportation activities. Data adapted from [24–27].

Type	Value	Unit
Virgin aggregate production	3.2	kg-CO_2/t-aggregate
Recycled aggregate production	3.2	kg-CO_2/t-aggregate
Concrete mixing and batching	1.2	kg-CO_2/t-concrete
Concrete onsite placement	5.6	kg-CO_2/t-concrete
Cement transportation	4.4	kg-CO_2/t-cement
Aggregate and SCMs transportation	8.1	kg-CO_2/t-aggregate
Concrete transportation	1.1	kg-CO_2/t-concrete
Buried aggregate transportation	8.1	kg-CO_2/t-aggregate

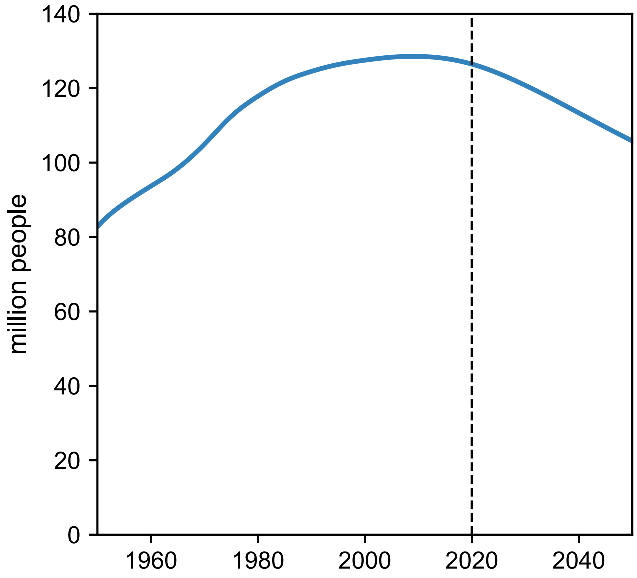

Fig. S11 Population projections data, 1950-2050. Data are obtained from the shared socioeconomic pathways 2, which represents a middle-of-the-road scenario [31].

Table S15 Summary of variables and parameters of a physicochemical model [3,4] to quantify the CO_2 uptake from concrete carbonation.

Item	Value	Unit	Ref.
Shares of cement used in concrete	79.3	%	[20]
Shares of cement used in mortar	20.7	%	[20]
Market shares of concrete strength classes	Table S16	%	[32]
Shares of mortar uses	Table S17	%	[3]
Proportion of CaO converted to $CaCO_3$	Table S18	%	[4]
Carbonation rates of concrete	Table S19	$mm/\sqrt{yr}$	[4]
Carbonation rate of mortar	Table S20	$mm/\sqrt{yr}$	[3]
Correction factor	Table S21	-	[4]
Thickness of concrete uses	Table S22	mm	[3]
Thickness of mortar uses	Table S23	mm	[3]
Exposure time during the demolition stage	0.2	year	[33]
Breakdown of demolition waste by particle size	Table S24	%	[3]
CaO content in clinker	65.8	%	[18]
CaO content of CKD	44.0	%	[4]
Mole ratio of CO_2 to CaO	0.785	-	-

Table S16 Market shares of concrete strength classes. Data adapted from [32].

Strength classes	Distribution	Scale	Shape
≤C15	Weibull	0.0%	12.0
C16-C23		35.5%	12.0
C23-C35		41.3%	16.0
>C35		27.1%	12.0

Table S17 Shares of mortar uses. Data adapted from [3].

Use	Distribution	Scale	Shape
Rendering and plastering	Weibull	52.4%	14.0
Masonry		18.8%	12.0
Repairing and maintenance		33.2%	10.0

Table S18 Proportion of CaO converted to $CaCO_3$. Data adapted from [4].

Use	Distribution	Scale	Shape
Concrete	Weibull	86.0%	25.0
Mortar		92.0%	20.0

Table S19 Carbonation rates of concrete with different strength classes in different exposure conditions [Unit: mm/$\sqrt{\mathrm{yr}}$]. Data adapted from [4].

Exposure conditions	Strength class	Distribution	Max	Min
Indoor, outdoor exposed, and outdoor sheltered	≤C15	Uniform	15.0	5.0
	C16-C23		9.0	2.5
	C23-C35		6.0	1.5
	>C35		3.5	1.0
Buried	≤C15		3.0	2.0
	C16-C23		1.5	1.0
	C23-C35		1.0	0.8
	>C35		0.8	0.5

Table S20 Carbonation rates of mortar [Unit: mm/$\sqrt{\mathrm{yr}}$]. Data adapted from [3].

Item	Distribution	Mode	Max	Min
Carbonation rate of mortar	Triangular	19.6	36.8	6.1

Table S21 Correction factor of cement additives, CO_2 concentration, and coating and cover. Data adapted from [4].

Item	Distribution	Scale	Shape
Cement additives	Weibull	1.2	20.0
CO_2 concentration		1.2	25.0
Coating and cover		1.0	6.0

Table S22 Thickness of concrete uses [Unit: mm]. Data adapted from [3].

Item	Distribution	Max	Min
Concrete structures	Uniform	610.0	60.0

Table S23 Thickness of mortar uses [Unit: mm]. Data adapted from [3].

Mortar use	Distribution	Scale	Shape
Rendering and plastering	Weibull	22.0	4.0
Masonry		11.0	8.0
Repairing and maintenance		26.8	7.0

Table S24 Percentages of different particle sizes [Unit: %]. Data adapted from [3].

Secondary use	Particle size	Distribution	Max	Min
New concrete	<5 mm	Uniform	37.0	15.0
	5-10 mm		23.0	12.0
	10-20 mm		46.0	24.0
	20-40 mm		39.0	16.0
Road base	<1 mm		24.7	10.0
	1-10 mm		28.0	20.3
	10-30 mm		51.3	35.3
	30-53 mm		26.0	10.7
Landfill	<10 mm		25.6	12.2
	10-30 mm		35.4	19.5
	30-50 mm		22.5	10.6
	>50 mm		48.4	24.8
Asphalt concrete	<5 mm		37.0	15.0
	5-10 mm		23.0	12.0
	10-20 mm		46.0	24.0
	20-40 mm		39.0	16.0

3. Strategy

3.1 Strategy details

Table S25 Summary of supply- and demand-side strategies and their model implementation.

Classification	Strategy	Model implementation
Conventional supply-side strategies	Electricity decarbonization	100% share of non-emitting electricity
	Thermal efficiency improvement	3000 MJ/t-clinker
	Electrical efficiency improvement	85 kWh/t-cement
	Low-carbon fuel utilization	Use of 50% natural gas and 50% waste fuel
	Clinker-to-cement ratio reduction	Replacement of 50% of clinker with supplementary cementitious materials
	Low-carbon transportation	A 75% reduction in emissions from transportation activities
Emerging supply-side strategies	Lower-carbon cement chemistries	Use of lower-carbon cement chemistries in mortars and concrete of strength classes ≤C15 and C16-C23
	CCU (concrete curing)	A 34% adaptation rate in concrete production
	CCU (mineralization)	Replacement of virgin aggregate by synthetic aggregate made by reacting alkaline industrial waste with CO_2
Demand-side (material efficiency) strategies	Material-efficient design	A 15% reduction in cement content in concrete and a 13% reduction in concrete intensity of buildings and infrastructure
	Construction waste reduction	A 1% reduction in construction waste generation
	More intensive use	A 20% reduction in building floor space per capita and a 9% reduction in infrastructure stock per capita
	Lifetime extension	A 90% increase in building lifetime and a 30% increase in infrastructure lifetime
	Component reuse	A 15% reuse rate of demolished building concrete
	Downcycling	A 10% downcycling rate of demolished concrete
	Waste stockpiling	Extension of waste stockpiling period to one year

Electricity decarbonization

Decarbonization of the electricity supply will lead to a reduction in CO_2 emissions associated with electricity use, i.e., indirect emissions. This study assumes that the electricity supply will be decarbonized in line with the International Energy Agency's Sustainable Development Scenario through 2040 [23]. Further, a transition to a 100% share of non-emitting electricity is assumed for 2050.

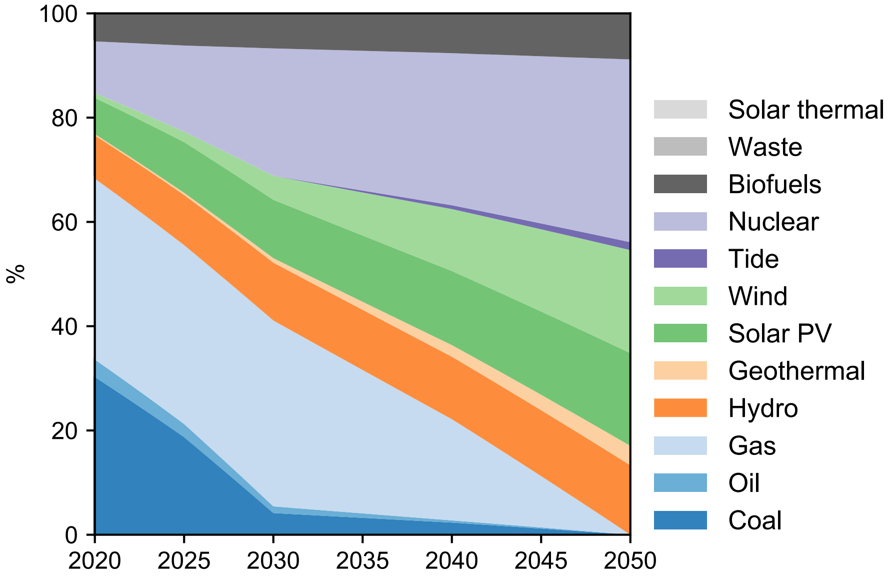

Fig. S12 Electricity generation mix, 2020-2050.

Thermal efficiency improvement

Clinker production in Japan has been shifting rapidly from wet kilns with inferior thermal efficiency and dry kilns without preheating equipment to dry kilns (SP kilns) with preheating equipment (preheaters) and NSP kilns with higher production efficiency since the 1960s, and the shift was completed by around 2000 [21]. Consequently, the thermal efficiency of clinker production in Japan is already close to the practical minimum and there is not much room for improvement. However, there are now plans to expand the use of more energy-efficient equipment, such as high-efficiency clinker coolers, which will improve the heat recovery efficiency compared to the conventional type [21]. Based on the average thermal efficiency of the global 10% best-in-class, this study assumes that the thermal efficiency can be improved up to 3000 MJ/t-clinker by 2050 [24].

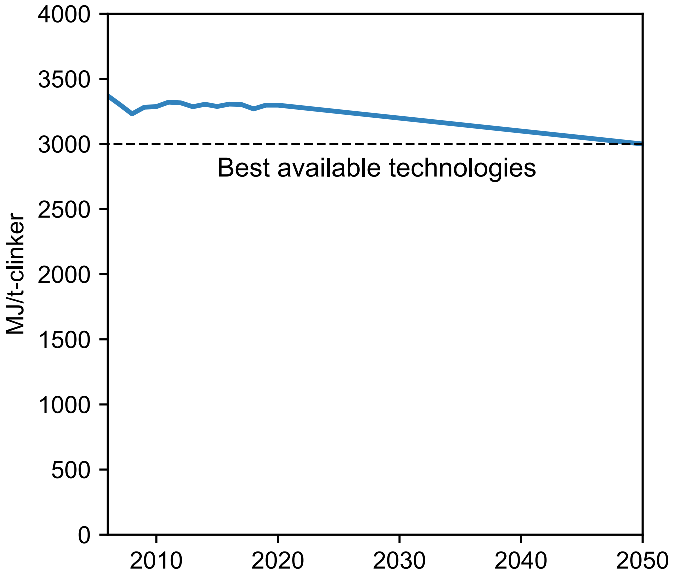

Fig. S13 Thermal efficiency in the cement kiln, 2006-2050.

Electrical efficiency improvement

In the cement production processes, electrical energy is required for various facilities, such as grinding mills, classifiers, air blowers for clinker cooling, and rotary kiln operation. Since increasing electrical efficiency by energy recovery through, for example, thermal energy, energy conservation in the industry is being achieved by increasing the efficiency per unit of electrical energy [21]. This study assumes that the electrical efficiency can be improved up to 85 kWh/t-cement by 2050 by employing highly advanced single-particle comminution grinding technology [24].

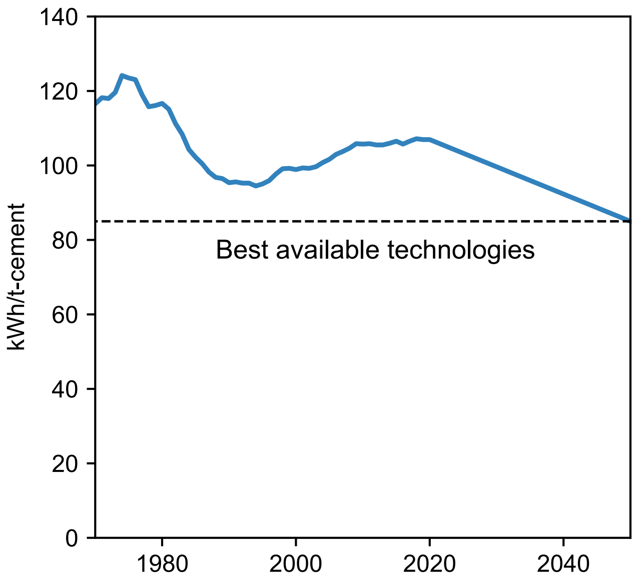

Fig. S14 Milling/grinding electrical efficiency, 1970-2050.

Low-carbon fuel utilization

The main fuel used to fire kilns has historically been coal, due to its widespread availability, high calorific value, and generally low cost [7]. The Japanese cement industry has a long-term vision to completely switch to natural gas and waste fuels in order to reduce CO_2 emissions from fuel combustion [34]. This study assumes that the shares of natural gas and waste fuels (assuming waste plastics) in thermal energy generation can be increased from current levels to 50% each by 2050. This means that there will be a complete shift away from coal in fuel combustion by 2050. The emission factor for each fuel follows the national emission factor shown in **Table S12**. Note that the emission factor only accounts for emissions associated with fuel combustion and does not include leakage from fossil fuel mining, processing, transportation, storage, and other processes. Currently, due to the small scale of domestic production in Japan, emissions from fuel leakage in the natural gas supply chain account for only about 0.1% of total emissions [35], but this figure increases significantly when imports are taken into account [36]. Therefore, when expanding the use of natural gas in cement production, it is important to minimize fuel leakage throughout the natural gas supply chain.

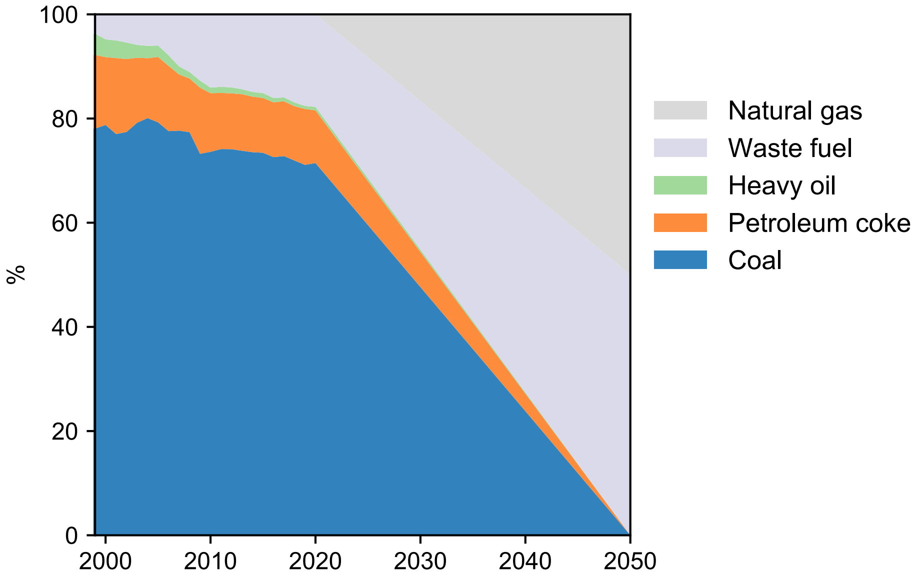

Fig. S15 Fuel mix of thermal energy consumption, 1999-2050.

Clinker-to-cement ratio reduction

Since most of the CO_2 emitted during the cement production process comes from clinker production, reducing the clinker-to-cement ratio can contribute to emission savings. Clinker can be replaced by a variety of supplementary cementitious materials (SCMs), such as fly ash, ground granulated blast furnace slag (GGBFS), and calcined clay [37]. In Japan, GGBFS and fly ash are mainly standardized and used. However, the availability of these industrial by-products depends on steel production and coal-fired power generation, and their availability is expected to decrease in a net-zero future. Therefore, we focus on calcined clays, especially in combination with limestone (i.e., LC^3 technology). Since the LC^3-50 blend (50% clinker, 30% calcined clay, 15% limestone and 5% gypsum) has already proven to be capable of providing mechanical properties comparable to standard ordinary Portland cement as well as provide some durability improvements [38], this study adopts an aggressive target of 50% clinker replacement by 2050 [39]. Although the substitution ratio of SCMs to clinker can vary with compressive strength, workability, or exposure conditions, a one-to-one substitution ratio is assumed here, as in several existing studies [24,40].

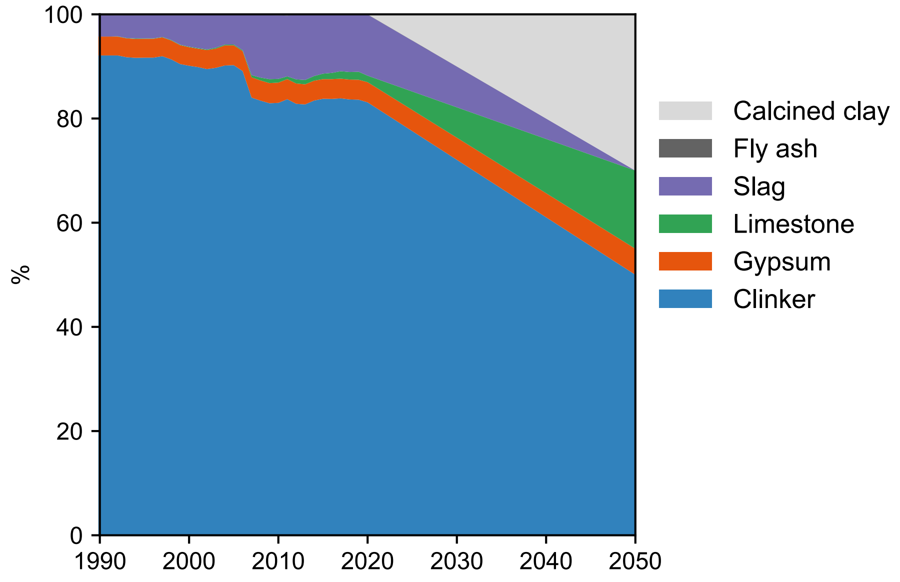

Fig. S16 Cement ingredients, 1990-2050.

Low-carbon transportation

In the 'Modern Truck Scenario' proposed by the International Energy Agency [41], systemic improvements in operations and logistics across all aspects of road freight, increased vehicle efficiency, and support for the use of alternative fuels in transport are expected to reduce well-to-wheel greenhouse gas emissions by around 75% compared to the baseline scenario. Based on this evidence, this study assumes a 75% reduction in CO_2 emissions intensity from the transport of cement, aggregates, and concrete by 2050.

Low-carbon cement chemistries

Several low-carbon cement chemistries, such as reactive belite cement, can be produced in conventional cement kilns. However, due to relatively high raw material costs, lack of reliable test methods, and lack of product standardization, these chemistries are currently limited to niche markets [42]. Although research on these cement chemistries has increased in recent years, their durability when used in concrete remains poorly understood [43]. We therefore assume that the application of low-carbon cement chemistries is limited to mortar and low compressive strength concrete (primarily for non-structural applications) [24]. Based on the proportion data for mortar and strength classes ≤C15 and C16-C23, the maximum applicable proportion of low-carbon cement is assumed to be 55%. Due to data limitations, this study assumes a uniform increase in the ratio of each low-carbon cement chemistry by 2050. The emission and energy savings of each chemistry are shown in **Table S26**.

Table S26 Process CO_2 and energy savings of low-carbon cement clinker compared to OPC clinker [Unit: %]. The alkali reduction rate is also presented for modeling the impact on carbonation. Data adapted from [24,44].

Type	Process CO_2 savings	Thermal energy savings	Alkali reduction
Reactive belite cement	3.1	8.2	3.1
Belite-ye’elimite-ferrite cement	29.1	34.9	24.1
Carbonatable calcium silicate cement	24.8	38.9	100.0
Calcium sulfoaluminate cement	42.0	46.9	33.5
Celitement	33.2	50.6	33.2
Magnesium oxides derived from magnesium silicates	100.0	46.5	43.0

CCU (concrete curing)

Concrete curing with CO_2 refers to an accelerated carbonation process in which CO_2 gas is injected more thoroughly during the batching and mixing of concrete or during the curing process of precast products. This technology can reduce the CO_2 emissions associated with the concrete cycle in two ways: increased CO_2 uptake through accelerated carbonation, and savings in binders to achieve the required compressive strength [45]. However, because of the risk of corroding the steel frame inside, the concrete produced using this technology is difficult to use for some applications such as for reinforced concrete buildings [46]. However, concrete produced in this way is well suited for use in exterior materials employed in the civil engineering field, such as paving blocks and fence foundations [47]. Accordingly, we assume a maximum application rate of 34%, based on the strength class data. In calculating emission savings, this study assumes the following [24]: (1) the energy penalty from CO_2 transport and injection is 0.3 kg-CO_2/t -concrete; (2) the total increase in CO_2 uptake is approximately 12% over the service life of the concrete; (3) binder reduction for both ready-mixed and precast concrete due to increased concrete compressive strength is approximately 13%.

CCU (mineralization)

Mineralization is a technology that absorbs CO_2 by exploiting the property of substances containing elements such as calcium and magnesium to become carbonate minerals upon contact with CO_2. The carbonates produced by this technology include calcium carbonate, potassium carbonate, and magnesium carbonate. Among these, calcium carbonate can be used as an aggregate for concrete, and a promising source of raw calcium is through recovery from industrial waste [46]. Therefore, we assume that 1% and 0.5% of concrete production will be able to use synthetic aggregates made from blast furnace slag and lime mud, respectively, by 2050 [24]. The use of fly ash and red mud is not considered, because coal-fired power generation is assumed to be phased out and Japan relies on imports for 100% of its aluminum ingots. Blast furnace slag should also decline as steel production is decarbonized, but the industry roadmap shows that some blast furnaces will remain in 2050 [48]. Further, Japan's CCU roadmap specifies the utilization of blast furnace slag [47]. Consequently, we assume that the utilization of blast furnace slag for this technology is viable. In addition, we assume that 10% of demolished concrete can be converted into synthetic aggregates with this technology, with a 12% increase in CO_2 uptake over the service life of the concrete, which is similar to concrete curing [24]. The same energy penalty is assumed as for concrete curing [24]. Although the effect of the use of synthetic aggregates on the compressive strength of concrete is not well known, we assume that there is no particular effect.

Table S27 Parameter summary of CCU mineralization using alkaline industrial waste. Data adapted form [24].

Type	CO_2 uptake	Unit
Blast furnace slag	259.6	kg-CO_2/t-blast furnace slag
Lime mud	70.0	kg-CO_2/t-lime mud

Material-efficient design

Performance-based design allows architects and contractors to design concrete mixtures that meet the necessary mechanical and durability specifications with less cement. Some evidence suggests that the cement content of concrete could be reduced by 15-20% without compromising compressive strength [49,50]. Importantly, a previous study showed that prescriptive design, which specifies the allowable cementitious content in concrete, induces over-use of cement [51]. According to their estimates, using performance-based design rather than prescriptive design could reduce carbon emissions per required volume of concrete by up to 30%. Here, we assume that the cement content of concrete can be reduced by 15% by 2050 [40]. Furthermore, precast components allow designers to manufacture concrete components with greater precision and reliability using less cement. Post-tensioning techniques can make parts of concrete elements thinner by stressing the rebar in the concrete floor slab before applying external loads. Changes in the way elements are specified could reduce the amount of cementitious materials used in structural components, especially as overdesign is often employed by designers when constructing structural elements. Together, we assume that a 13% reduction in the concrete intensity of concrete structures can be achieved without interfering with its function by 2050 [24,40]. Currently, the concrete intensity of buildings in Japan is two to four times higher than in the U.S. and China. This difference is mainly due to the structure of the building, especially the thickness of the foundation, piles, and columns [52]. Since the strategies considered here do not compromise strength or durability of structures, we assume that transitions can be encouraged by making adjustments to building codes and through appropriate code enforcement.

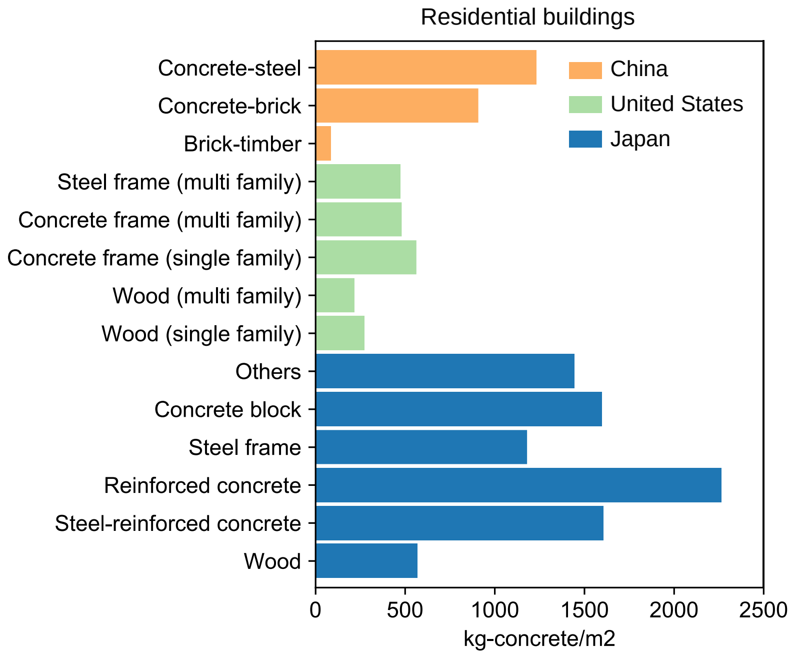

Fig. S17 International comparison of concrete intensity of newly constructed residential buildings. Data adapted from [24].

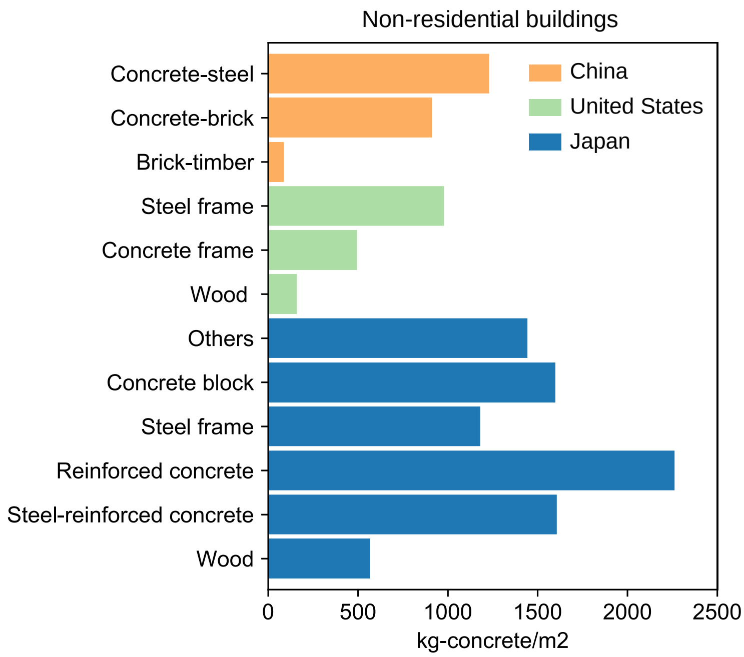

Fig. S18 International comparison of concrete intensity of newly constructed non-residential buildings. Data adapted from [24].

Construction waste reduction

There are opportunities to improve fabrication yields by targeting wasteful activities, such as excessive cement orders at the construction stage [40,53]. Material losses during construction can be avoided by improving the architectural and engineering specifications of cement or by using excess cement (in cases where the amount of cement used in a project turns out to be less than the amount ordered) for other purposes. The potential also exists to reduce construction waste by promoting prefabrication and digitalization. This study assumes a 1% improvement in construction waste generation rates through these efforts by 2050 [53].

More intensive use

More intensive use refers to reducing the total building floor space and infrastructure required to provide the same level of service through such means as enhanced sharing practices and urban structural changes. For residential buildings, this study assumes a convergence of floor space per capita to 30 m^2/cap by 2050, referring to the Low Energy Demand scenario [54,55]. This corresponds to a reduction of approximately 20% from current levels. For non-residential buildings, we also assume 20% reductions from current levels by 2050 [24,56]. More intensive use of infrastructure can be achieved through the centralization of urban functions [57]. Much of Japan's infrastructure was built in the 20th century, and plans for centralization and downsizing of aging infrastructure are under discussion. The primary motivation for this is to reduce the cost of maintaining aging infrastructure due to a shrinking and aging population, but it will also contribute to more intensive use. We assume a 9% reduction in infrastructure stock per capita from current levels through urban structural changes [58]. The implementation speed is modeled by a gradual curve to avoid abrupt changes [55].

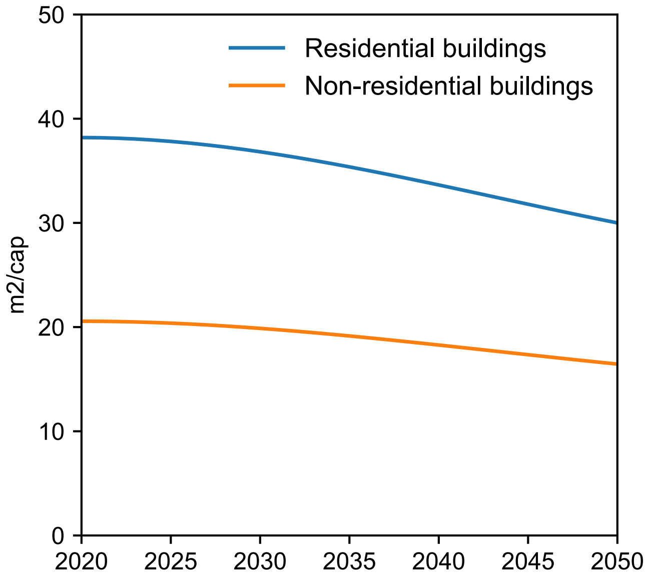

Fig. S19 Building stock per capita in Japan, 2020-2050.

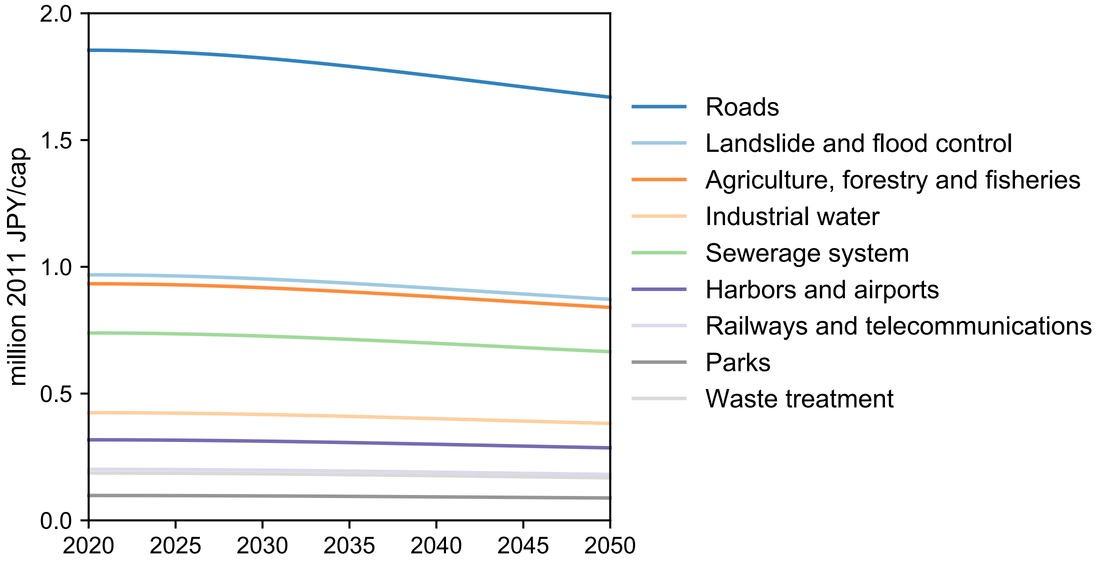

Fig. S20 Infrastructure stock per capita in Japan, 1950-2020.

Lifetime extension

Extending the service life of buildings and infrastructure can curb demand for new construction activities. In Japan, the 'Act for Promotion of Long-Life Quality Housing' was enacted in 2008 to encourage the extension of the service life of housing through economic incentives. Further, ministries and agencies have formulated action plans for extending the service life of infrastructure facilities under their jurisdiction based on the 'Basic Plan for Extending the Service Life of Infrastructure'. Key measures include early detection of deterioration and damage through periodic inspections, development of electronic maintenance information, and use of sensors and robots in inspections and repairs. Based on the literature [59,60], this study assumes that the service life of buildings and infrastructure can be extended by 90% and 30% from current levels, respectively. Relatively conservative assumptions are made that the lifetime buildings and infrastructure that are constructed after 2021 will be extended, which means that the lifetime extension of existing stock is not considered. Increased maintenance for extended lifetime may involve additional carbon emissions, but that is not considered here due to lack of reliable data.

Component reuse

Modularized components can be reused in new construction projects, thus avoiding the production of concrete for new products. This strategy is possible through reversible or cyclic design (e.g., design for disassembly) together with a comprehensive database describing physical properties, previous use locations, loads, and environmental conditions [61]. We assume that 15% of demolished buildings can be reused by 2050 [56]. In this case, we assume that the reuse of components through reversible or cyclical design does not result in extra energy consumption for the dismantling of demolished buildings, or the transportation of new building materials.

Downcycling

Demolished concrete can be recycled to replace virgin aggregate in new concrete. The use of recycled aggregate can avoid CO_2 emissions associated with the mining and transportation of virgin aggregate [62]. This study assumes that the downcycling rate of demolished concrete can be increased to 10% by 2050 [24].

Waste stockpiling

During demolition, concrete is crushed into small pieces, which increases the surface area and promotes the carbonation process. Crushed concrete pieces are stored in intermediate treatment facilities for an average of 0.2 years in Japan [33]. Extending this storage period will promote the CO_2 absorption process of demolished concrete since the surface area of crushed concrete exposed to air is limited in a landfill environment [63]. The progress of carbonation of demolished concrete is estimated according to Fick's law of diffusion. In this case, we simply assume a longer stockpiling period and do not consider active systems, such as particle size refinement or wet-dry cycles that promote faster carbonation. Carbonation of demolished concrete proceeds faster with smaller particle sizes, as the area in contact with air is more extensive [64]. In addition, repeated drying and wetting promote faster carbonation due to water intervention [65]. While particle size refinement or wet-dry cycles can expedite carbonation, a key barrier to adopting these two measures at a large scale is the cost of transporting crushed concrete pieces to treatment facilities [24]. Compared to the aforementioned active measures, extending the stockpiling duration entails less transportation, but the maximum length of time that crushed concrete pieces can stay stockpiled is dependent on social acceptance and local regulations concrete. For example, the EU Construction and Demolition Waste Management Protocol and Guidelines suggest that the maximum stockpiling time is limited to one year [24]. Therefore, this study assumes that the stockpiling period of demolition waste can be extended to one year by 2050.

3.2 Interactions among demand-side strategies

It is important to note that the demand-side (material efficiency) strategies considered here are not independent and that they influence each other. A prominent example is the strategies related to lifetime extension and end-of-life options: the longer a concrete structure lasts through lifetime extension, the less demolition waste is available for reuse, downcycling, or waste stockpiling. Our model captures such interactions through a set of mass balance equations that ensure the feasibility of each strategy in terms of mass balance constraints.

However, there are also interactions among strategies that do not depend on mass balance constraints. For example, optimizing concrete components for material-efficient design may reduce the potential for component reuse, which requires standardization [60]. In addition, more intensive use may inhibit service life due to premature deterioration associated with high frequency of use of concrete structures [66]. Clearly, further research is needed on the relative quantitative influence of factors on the model parameters associated with such interactions among strategies.

In addition, interactions beyond the system boundary of the cement and concrete cycle are also important. For example, building longevity will slow the spread of better insulated buildings unless there are initiatives to retrofit existing buildings [67]. On the other hand, reductions in per capita floor space demand through space sharing and teleworking practices have the synergistic effect of reducing energy use during the utilization phase [59]. Modeling the synergistic effects of such interactions is another important task that needs to be undertaken in the future.

3.3 Barriers to strategy implementation

Table S28 Barriers in the implementation of several supply- and demand-side strategies. Based on a critical review of 37 previous studies [68], those mentioned more than once are checked.

		Energy efficiency improvement	Low-carbon fuel utilization	Clinker-to-cement ratio reduction	Low-carbon cement chemistries	CCUS	Material efficiency improvement
Economy	Higher cost	✓	✓	✓	✓	✓	✓
	Availability of materials		✓	✓	✓		
	Market uncertainty					✓	
	Lack of demand	✓		✓	✓	✓	✓
	Market acceptance			✓	✓		
	Fragmented supply chain						✓
Technical	Lack of infrastructure		✓			✓	
	Lack of expertise	✓					
	Poor information	✓					✓
	Additional energy requirements					✓	
	Time constraints						✓
	Longer curing time			✓	✓		
	Structural strength			✓			
Regulatory	Regulatory requirements		✓				✓
	Complex bureaucracy		✓			✓	
	Risk concern						✓
	Lack of effective policies			✓		✓	✓
	Institutional challenges	✓		✓			
Social	Social acceptance		✓			✓	
	Industry culture						✓

4. Additional results

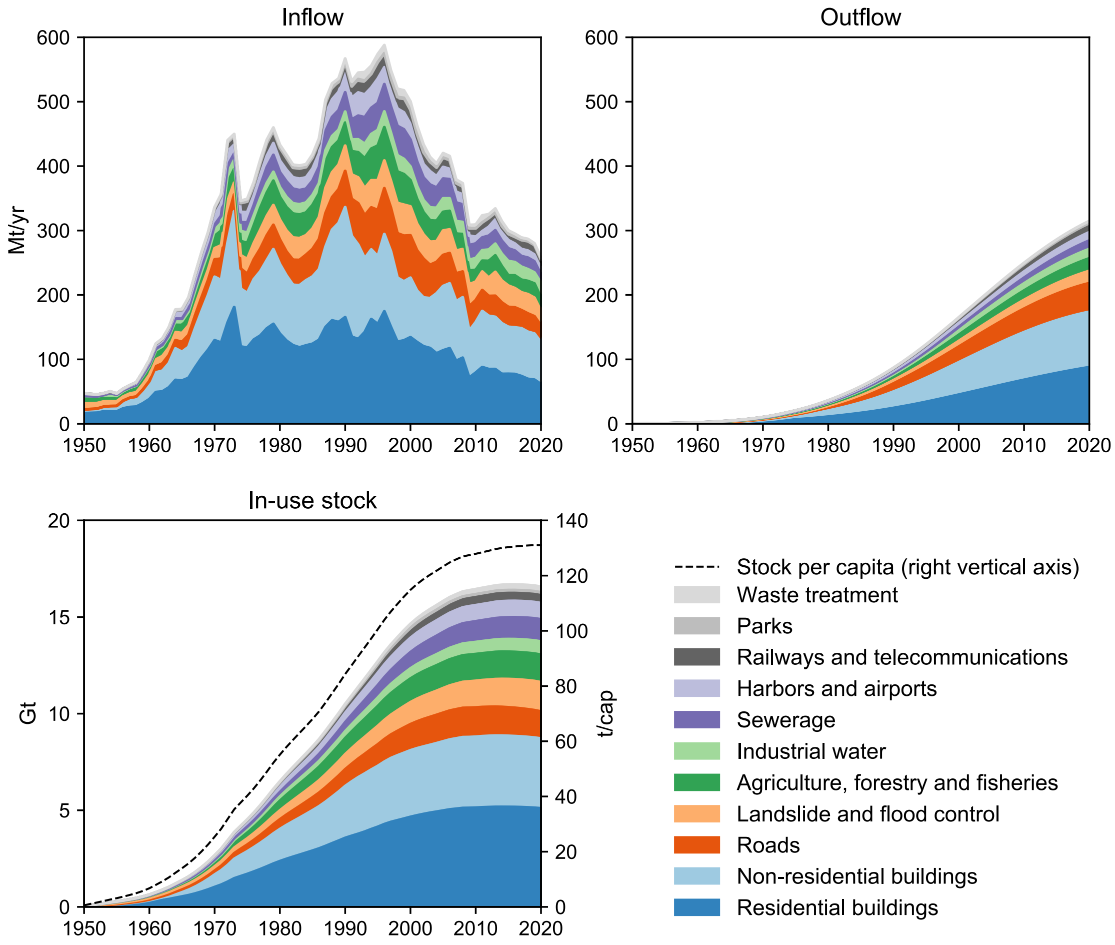

Fig. S21 Inflow, outflow, and in-use stock of concrete in Japan, 1950-2020.

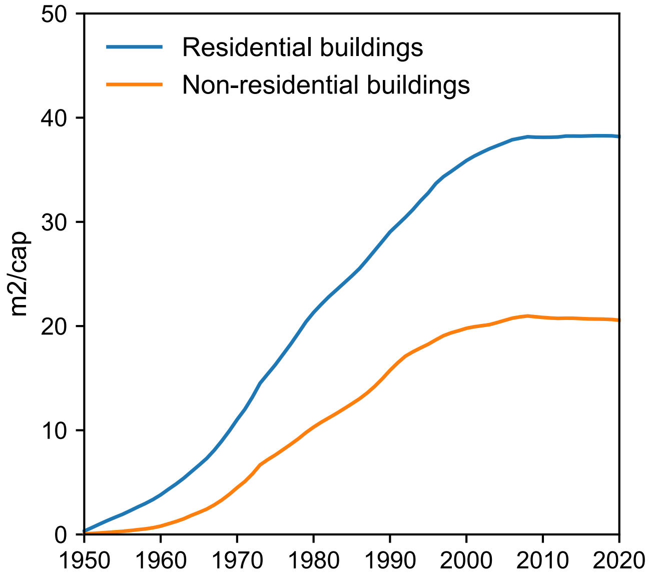

Fig. S22 Building stock per capita in Japan, 1950-2020.

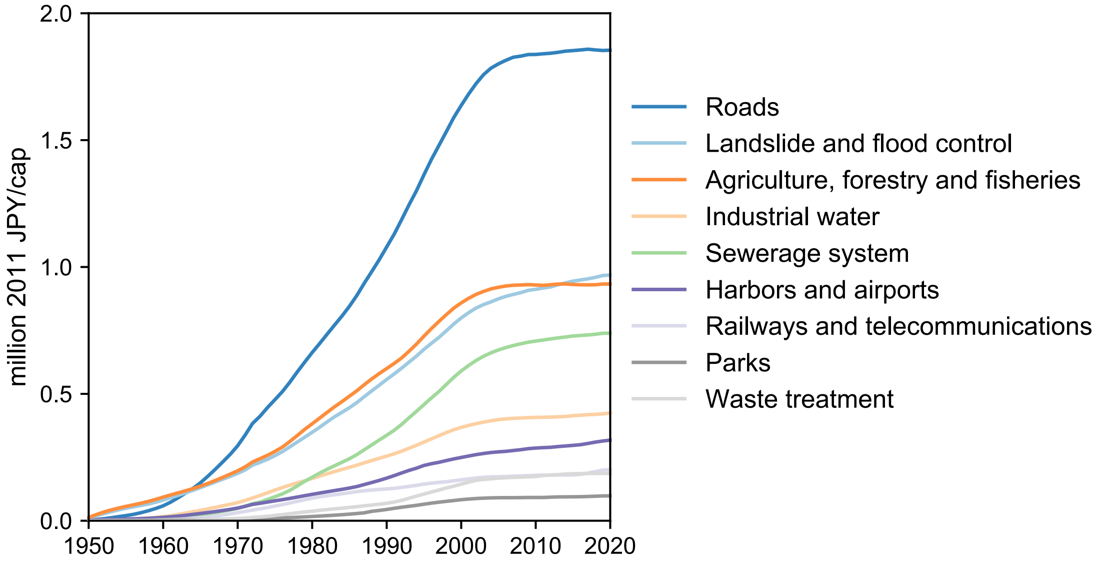

Fig. S23 Infrastructure stock per capita in Japan, 1950-2020.

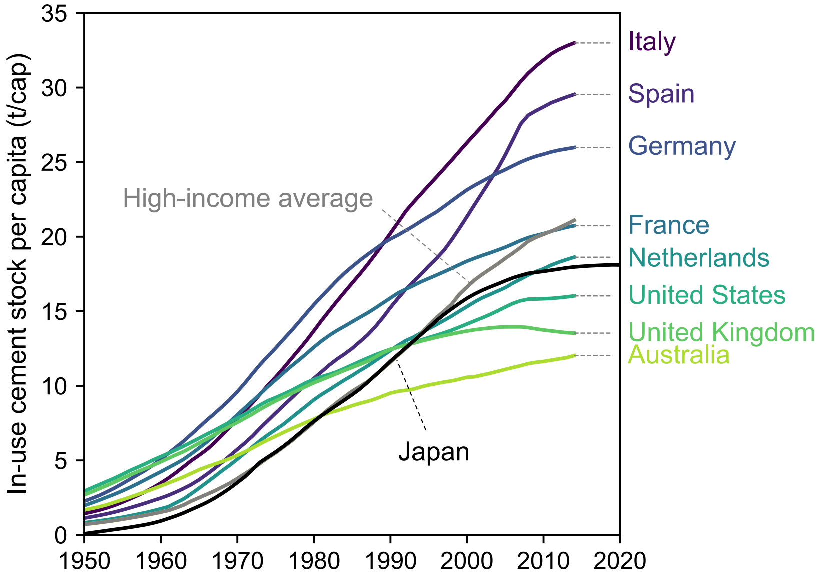

Fig. S24 International comparison of in-use stock of cement equivalent, 1950–2020. Data for countries other than Japan are based on our previous study [15], and country selection is based on other material flow analysis studies [69].

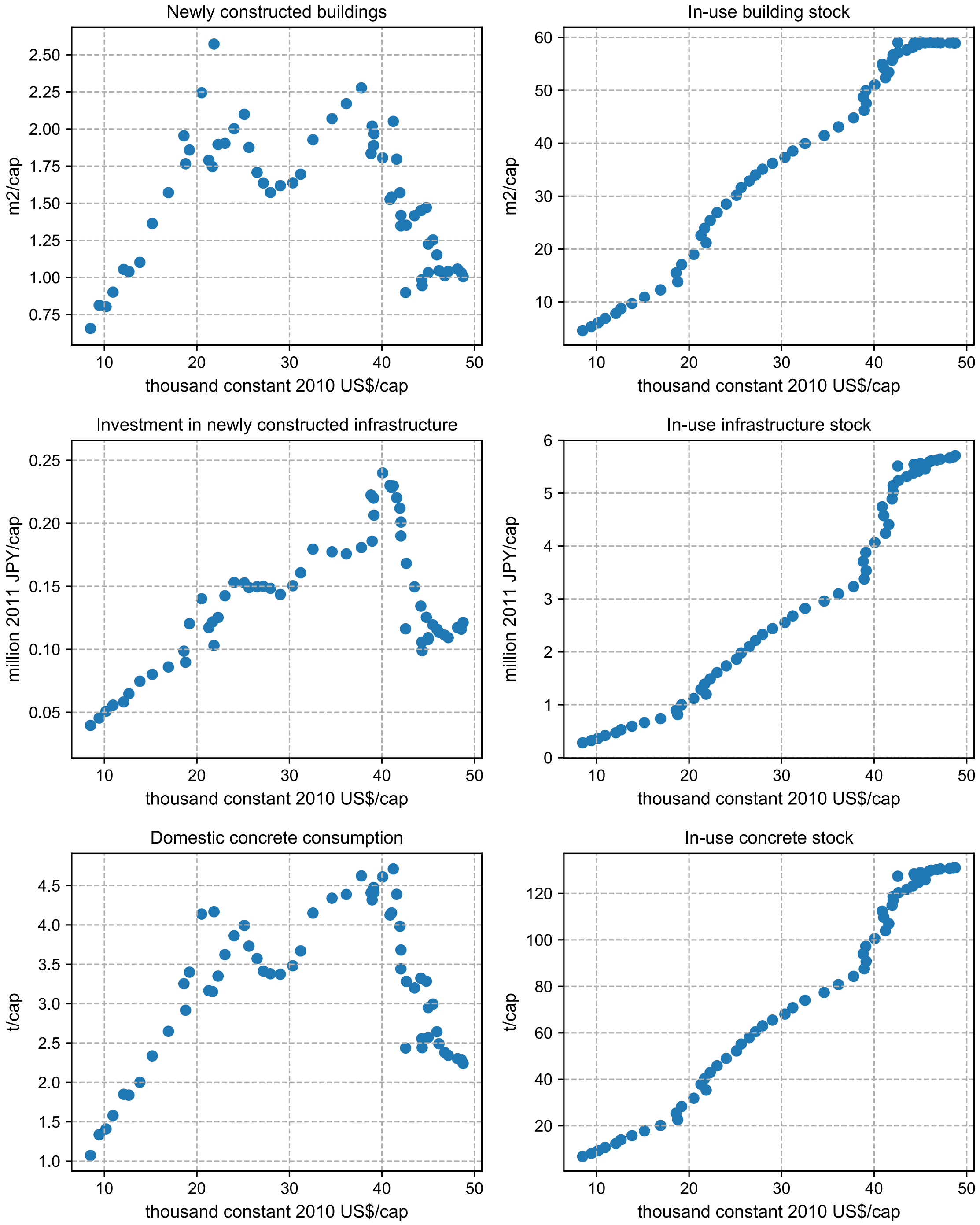

Fig. S25 Relationship between per capita GDP and key variables related to the cement and concrete cycle.

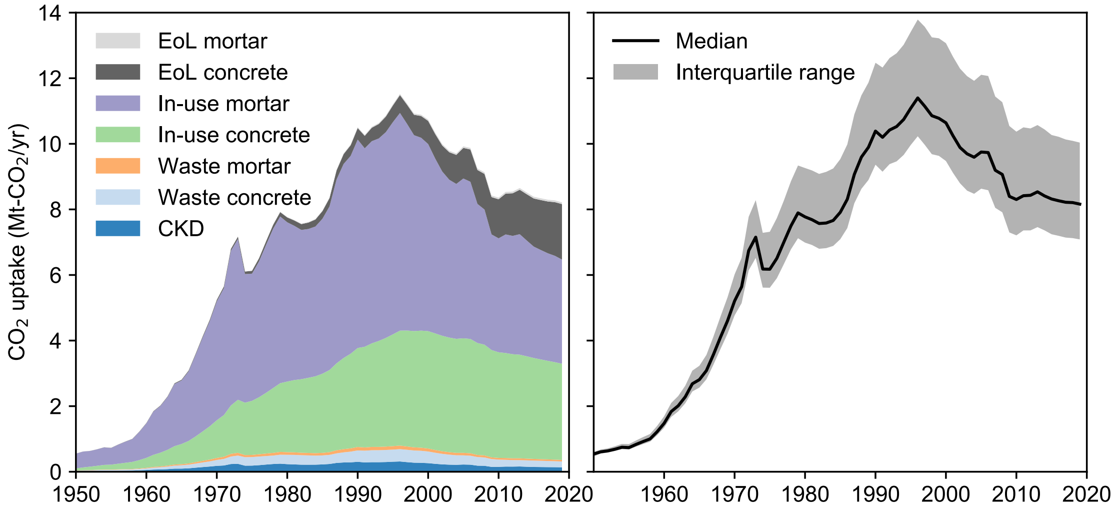

Fig. S26 CO_2 uptake from concrete carbonation, 1950-2019.

Note: CO_2 uptake has decreased for the last 30 years, in line with cement production trends. Such linkage is mainly due to in-use mortars. When cement is used as mortar, carbonation proceeds faster than in concrete due to its formulation and because it has a larger contact area with the atmosphere than concrete. Therefore, uptake from in-use mortars is strongly linked to the cement production trend, which has led to a downward trend in total uptake. Such trends are generally consistent with observations in previous studies [4,5].

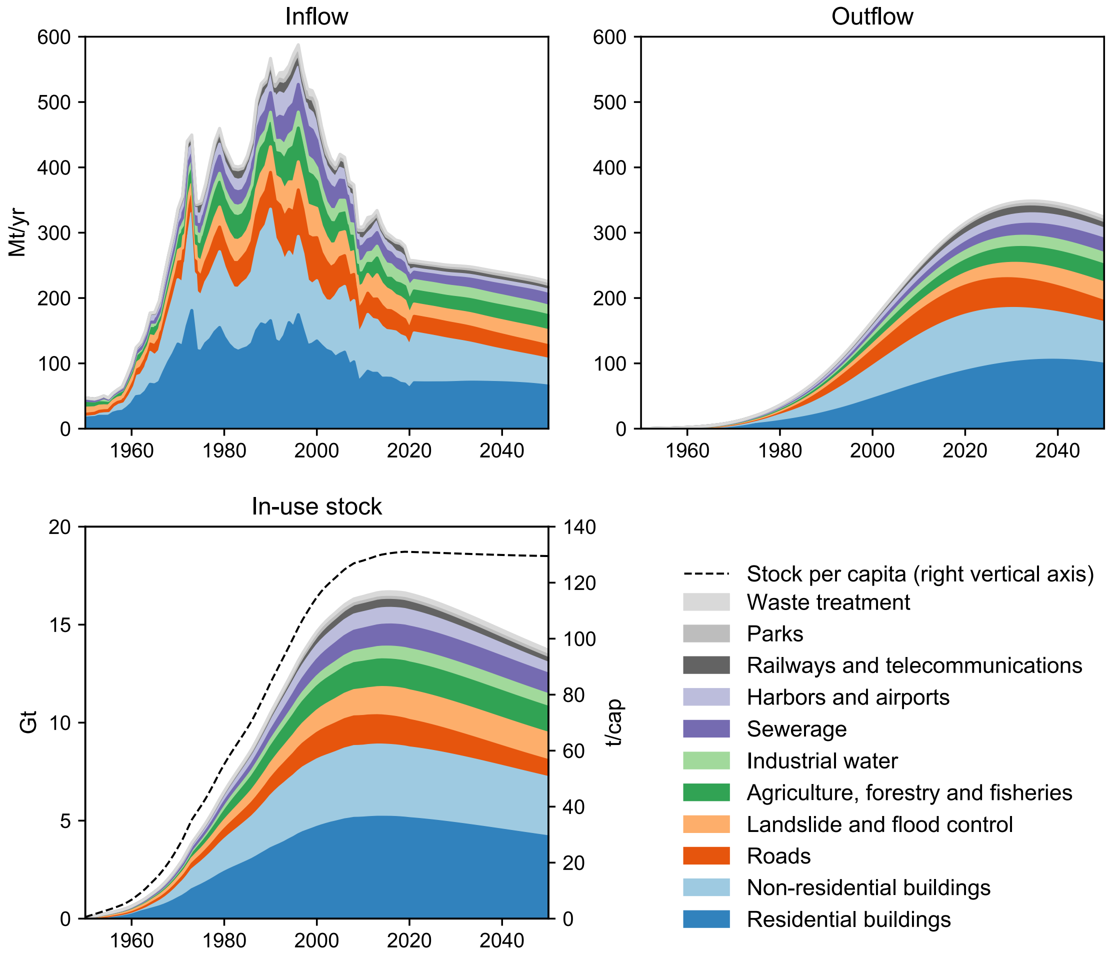

Fig. S27 Inflow, outflow, and in-use stock of concrete in Japan under the baseline scenario, 1950-2050

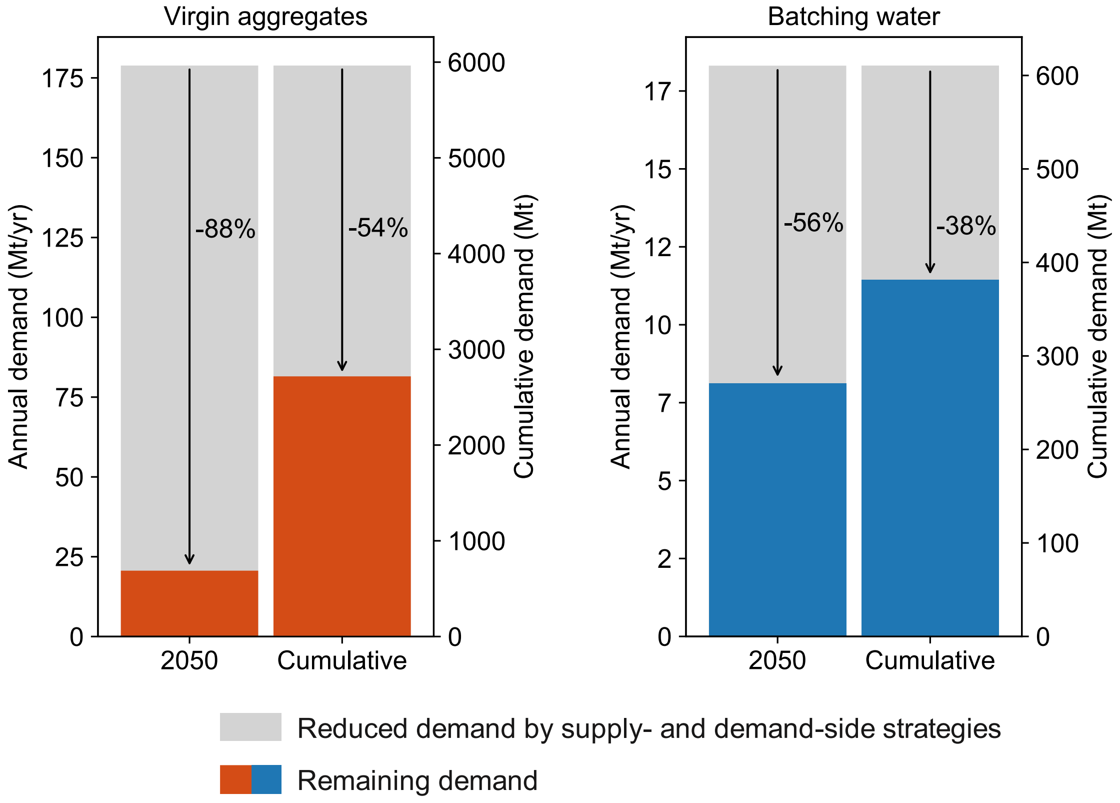

Fig. S28 Effects of supply- and demand-side strategies on virgin aggregates and batching water demand in Japan.

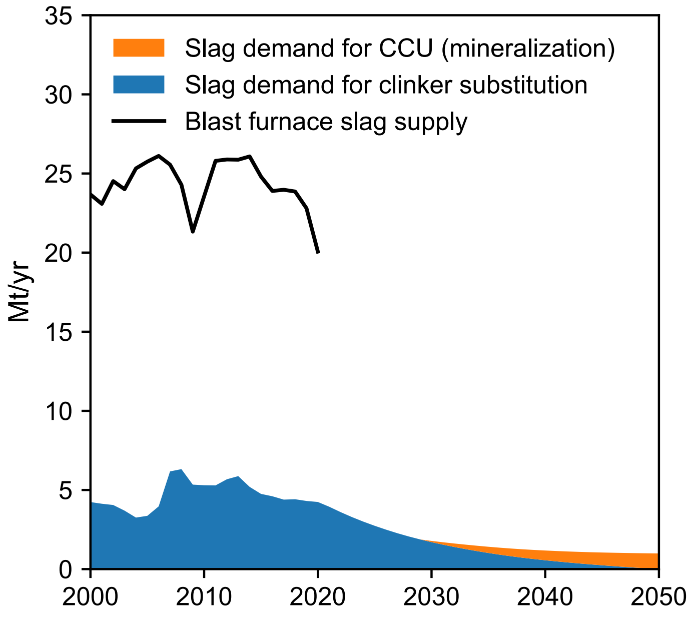

Fig. S29 Supply-demand balance of blast furnace slag in Japan when all strategies are fully implemented, 2000–2050. The gap between supply and demand includes use for exports, civil engineering, and construction. Data adapted from [70].

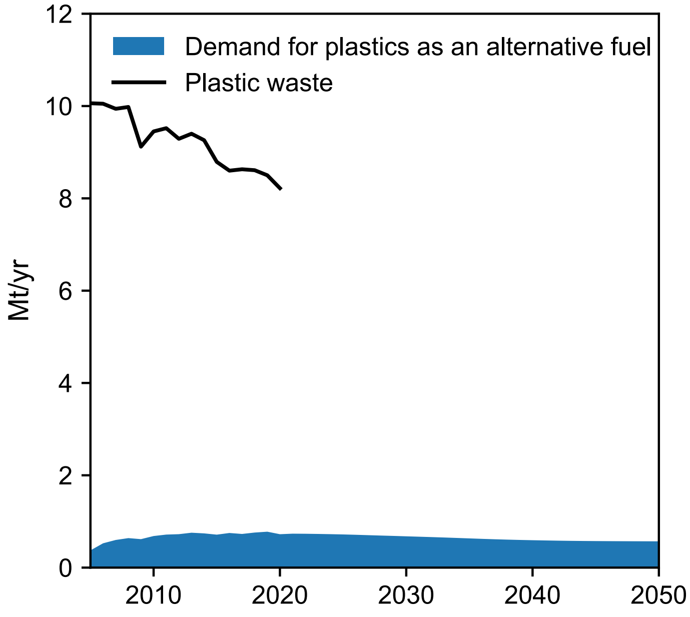

Fig. S30 Supply-demand balance of waste plastic in Japan when all strategies are fully implemented, 2005-2050. Data adapted from [71].

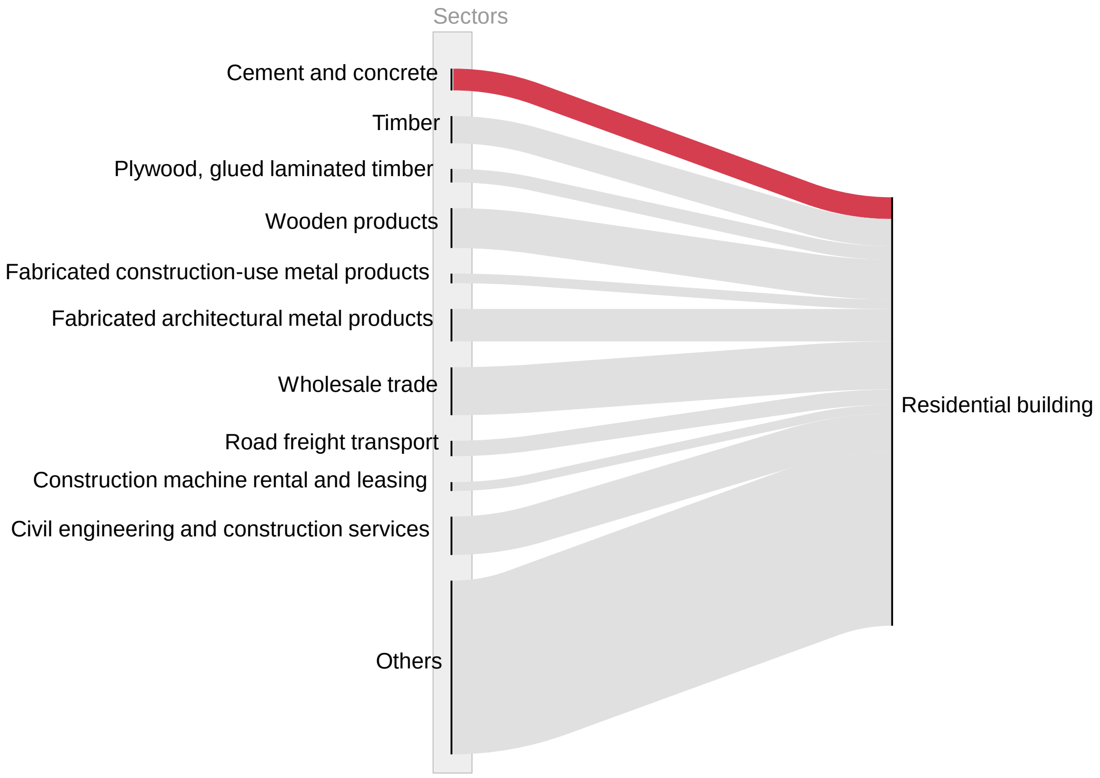

Fig. S31 Cement and concrete purchases as a percentage of total expenditures in the residential building construction sector. Data adapted from the Japanese Input-Output table [72].

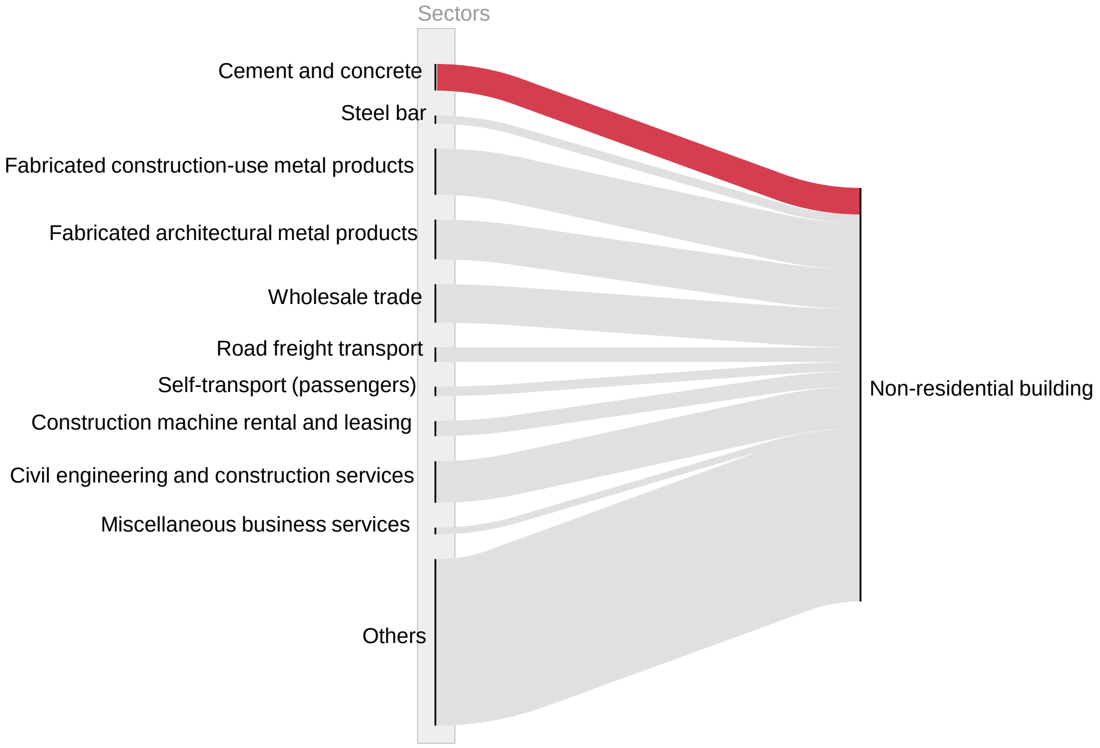

Fig. S32 Cement and concrete purchases as a percentage of total expenditures in the non-residential building construction sector. Data adapted from the Japanese Input-Output table [72].

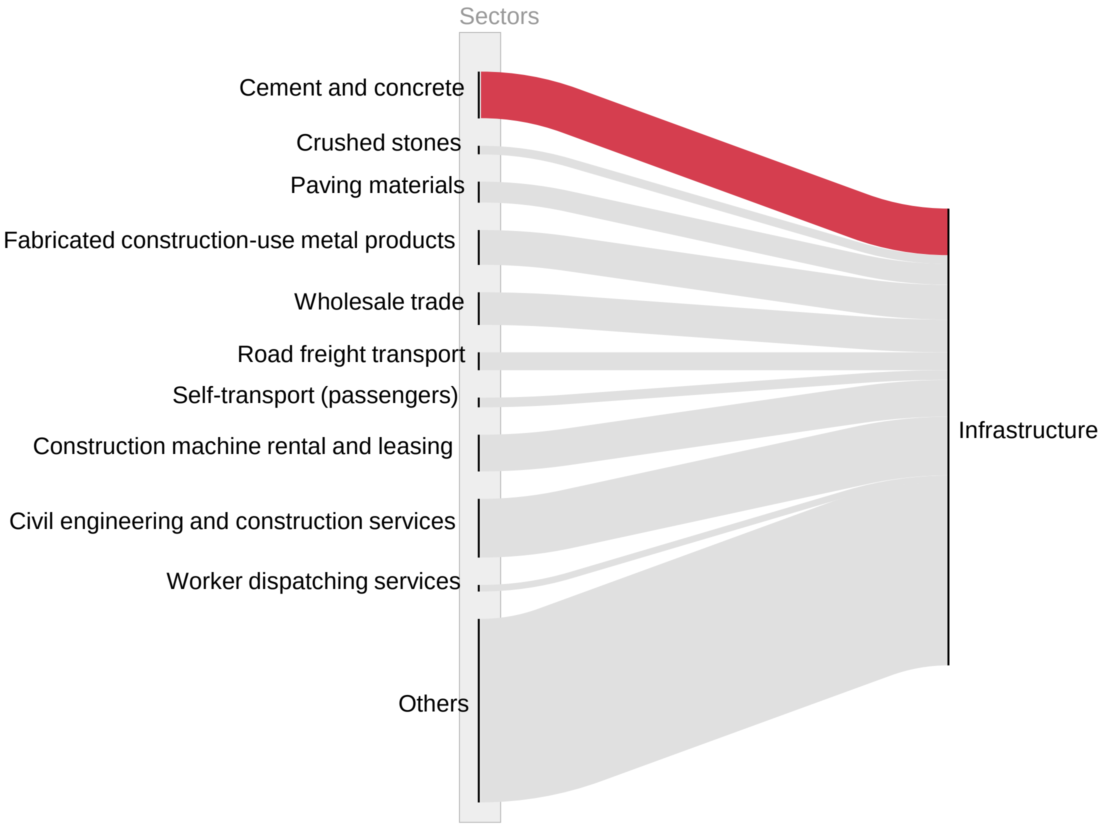

Fig. S33 Cement and concrete purchases as a percentage of total expenditures in the infrastructure construction sector. Data adapted from the Japanese Input-Output table [72].

5. Validation

This section provides a validation of our estimated results (**Fig. S34**). We can confirm that estimates of domestic cement consumption in this study are generally in good agreement with publicly available statistics. On the other hand, the estimates for cement and concrete stocks show some deviation from the two previous studies. This could be explained simply by the difference in the applications considered.

First, Cao et al. [15] considered all cement applications, including cementitious solidifiers, which are not covered in this study. The reason why cementitious solidifiers are not considered in this study is due to the model structure and incomplete data. Our model connects buildings and infrastructure construction activity information to the cement and concrete cycle. This process requires material intensity data for each construction activity, but the cement that is used as cementitious solidifiers is not included in the data collated by the government. We can assume that this is the prime reason for the larger estimates compared to those obtained in this study. Indeed, despite the difference in absolute values, the long-term trend itself is in good agreement with their findings, with stock growth becoming saturated from around 2000.

In contrast, Tanikawa et al. [29] estimated concrete stock volumes smaller than that estimated in this study, which may also reflect the difference in the applications considered. Specifically, the estimate of Tanikawa et al. [29] excludes landslide and flood control measures, agriculture, forestry and fisheries, and waste treatment. These applications account for approximately 20% of the total stock, which precisely explains the difference in the estimates.

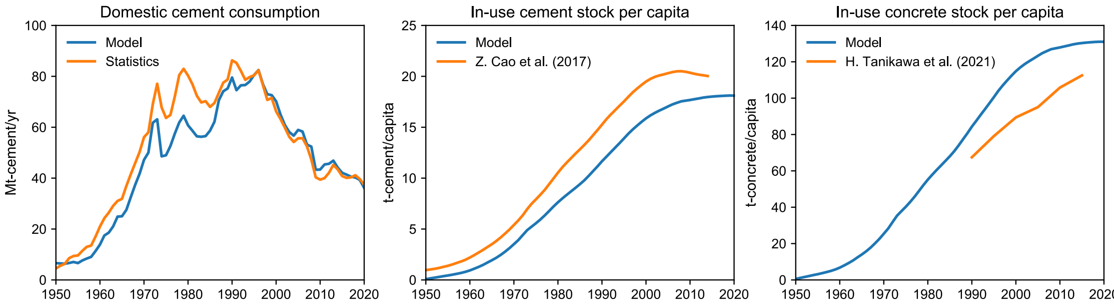

Fig. S34 Comparison of the estimates in this study with statistical data and previous studies.

6. Consumption categories in the input-output table

Table S29 Correspondences between the aggregated consumption categories and the sectors in the input-output table.

Consumption categories	Sector in the input-output table
Housing	Residential construction (wooden)
Housing	Residential construction (non-wooden)
Housing	House rent
Housing	House rent (imputed house rent)
Medical and health care	Medical service (hospitalization)
Medical and health care	Medical service (except hospitalization)
Medical and health care	Medical service (dentistry)
Medical and health care	Medical service (pharmacy dispensing)
Medical and health care	Medical service (miscellaneous medical service)
Medical and health care	Health and hygiene (public institution)
Medical and health care	Health and hygiene
Medical and health care	Social insurance
Medical and health care	Social welfare (public institution)
Medical and health care	Social welfare (private institution)
Medical and health care	Social welfare
Medical and health care	Nursery
Medical and health care	Nursing care (facility services)
Medical and health care	Nursing care (except facility services)
Transportation	Passenger motor cars
Transportation	Trucks, buses and miscellaneous cars
Transportation	Two-wheel motor vehicles
Transportation	Internal combustion engines for motor vehicles
Transportation	Motor vehicle parts and accessories
Transportation	Steel ships
Transportation	Miscellaneous ships (except steel ships)
Transportation	Internal combustion engines for vessels
Transportation	Repair of ships
Transportation	Rolling stock
Transportation	Repair of rolling stock
Transportation	Aircrafts
Transportation	Repair of aircrafts
Transportation	Bicycles
Transportation	Miscellaneous transport equipment
Transportation	Public construction of roads

Transportation	Railway construction
Transportation	Railway transport (passengers)
Transportation	Railway transport (freight)
Transportation	Bus transport service
Transportation	Hired car and taxi transport
Transportation	Road freight transport (except self-transport)
Transportation	Self-transport (passengers)
Transportation	Self-transport (freight)
Transportation	International shipping
Transportation	Coastal and inland water transport
Transportation	Harbor transport service
Transportation	Air transport
Transportation	Consigned freight forwarding
Transportation	Storage facility service
Transportation	Packing service
Transportation	Facility service for road transport
Transportation	Port and water traffic control (public corporation)
Transportation	Port and water traffic control
Transportation	Services related to water transport
Transportation	Airport and air traffic control (public corporation)
Transportation	Airport and air traffic control
Transportation	Services relating to air transport
Transportation	Travel agency and miscellaneous services related to transport
Transportation	Car rental and leasing
Transportation	Motor vehicle maintenance services
Education	School education (public institution)
Education	School education (private institution)
Education	Social education (public institution)
Education	Social education (private institution)
Education	Miscellaneous educational and training institutions (public institution)
Education	Miscellaneous educational and training institutions
Education	Research institutes for natural science (public institution)
Education	Research institutes for cultural and social science (public institution)
Education	Research institutes for natural sciences (private institution)
Education	Research institutes for cultural and social science (NPI)
Education	Research institutes for natural sciences
Education	Research institutes for cultural and social science
Education	Research and development (intra-enterprise)
Food	Rice
Food	Wheat, barley and the like

Food	Potatoes and sweet potatoes
Food	Pulses
Food	Vegetables
Food	Fruits
Food	Sugar crops
Food	Crops for beverages
Food	Miscellaneous edible crops
Food	Feed and forage crops
Food	Seeds and seedlings
Food	Flowers and plants
Food	Miscellaneous inedible crops
Food	Dairy cattle farming
Food	Beef cattle
Food	Hogs
Food	Hen eggs
Food	Chickens
Food	Miscellaneous livestock
Food	Silviculture
Food	Special forest products (including hunting)
Food	Marine fishery
Food	Marine aquaculture
Food	Inland water fishery and aquaculture
Food	Meat
Food	Dairy farm products
Food	Miscellaneous livestock products
Food	Frozen fish and shellfish
Food	Salted, dried or smoked seafood
Food	Bottled or canned seafood
Food	Fish paste
Food	Miscellaneous processed seafood
Food	Grain milling
Food	Flour and miscellaneous grain milled products
Food	Noodles
Food	Bread
Food	Confectionery
Food	Preserved agricultural foodstuffs
Food	Sugar
Food	Starch
Food	Dextrose, syrup and isomerized sugar
Food	Animal oil and fats, vegetable oil and meal
Food	Condiments and seasonings
Food	Prepared frozen foods

Food	Retort foods
Food	Dishes, sushi and lunch boxes
Food	Miscellaneous foods
Food	Refined sake
Food	Malt liquors
Food	Whiskey and brandy
Food	Miscellaneous liquors
Food	Tea and roasted coffee
Food	Soft drinks
Food	Manufactured ice
Food	School lunch (public institution)
Food	School lunch (private institution)
Food	Eating and drinking places
Food	Food take-out and delivery services
Utilities	Repair of construction
Utilities	Public construction of rivers, drainages and miscellaneous public construction
Utilities	Agricultural public construction
Utilities	Electric power facilities construction
Utilities	Miscellaneous civil engineering and construction
Utilities	Electricity
Utilities	Private power generation
Utilities	Gas supply
Utilities	Steam and hot water supply
Utilities	Water supply
Utilities	Industrial water supply
Utilities	Sewage disposal
Utilities	Waste management services (public corporation)
Utilities	Waste management services
Industrial products	Logs
Industrial products	Coal mining, crude petroleum and natural gas
Industrial products	Gravel and quarrying
Industrial products	Crushed stones
Industrial products	Miscellaneous ores
Industrial products	Feeds
Industrial products	Organic fertilizers, n.e.c.
Industrial products	Tobacco
Industrial products	Fiber yarns
Industrial products	Cotton and staple fiber fabrics (including fabrics of synthetic spun fibers)
Industrial products	Silk and artificial silk fabrics (including fabrics of synthetic filament fibers)
Industrial products	Miscellaneous fabrics

Industrial products	Knitting fabrics
Industrial products	Yarn and fabric dyeing and finishing (processing on commission only)
Industrial products	Miscellaneous fabricated textile products
Industrial products	Woven fabric apparel
Industrial products	Knitted apparel
Industrial products	Miscellaneous wearing apparel and clothing accessories
Industrial products	Bedding
Industrial products	Carpets and floor mats
Industrial products	Miscellaneous ready-made textile products
Industrial products	Timber
Industrial products	Plywood, glued laminated timber
Industrial products	Wooden chips
Industrial products	Miscellaneous wooden products
Industrial products	Wooden furniture
Industrial products	Metallic furniture
Industrial products	Wooden fixtures
Industrial products	Miscellaneous furniture and fixtures
Industrial products	Pulp
Industrial products	Paper
Industrial products	Paperboard
Industrial products	Corrugated cardboard
Industrial products	Coated paper and building (construction) paper
Industrial products	Corrugated card board boxes
Industrial products	Miscellaneous paper containers
Industrial products	Paper textile for medical use
Industrial products	Miscellaneous pulp, paper and processed paper products
Industrial products	Printing, plate making and book binding
Industrial products	Chemical fertilizer
Industrial products	Industrial soda chemicals
Industrial products	Inorganic pigment
Industrial products	Compressed gas and liquefied gas
Industrial products	Salt
Industrial products	Miscellaneous industrial inorganic chemicals
Industrial products	Petrochemical basic products
Industrial products	Petrochemical aromatic products (except synthetic resin)
Industrial products	Aliphatic intermediates
Industrial products	Cyclic intermediates, synthetic dyes and organic pigments
Industrial products	Synthetic rubber
Industrial products	Methane derivatives
Industrial products	Plasticizers
Industrial products	Miscellaneous industrial organic chemicals

Industrial products	Thermo-setting resins
Industrial products	Thermoplastics resins
Industrial products	High function resins
Industrial products	Miscellaneous synthetic resins
Industrial products	Chemical fibers
Industrial products	Medicaments
Industrial products	Oil and fat products and surface-active agents
Industrial products	Cosmetics, toilet preparations and dentifrices
Industrial products	Paint and varnishes
Industrial products	Printing ink
Industrial products	Agricultural chemicals
Industrial products	Gelatin and adhesives
Industrial products	Photographic sensitive materials
Industrial products	Miscellaneous final chemical products
Industrial products	Petroleum refinery products (including greases)
Industrial products	Coal products
Industrial products	Paving materials
Industrial products	Plastic products
Industrial products	Tires and inner tubes
Industrial products	Miscellaneous rubber products
Industrial products	Leather footwear
Industrial products	Leather tanning, leather products and fur skins (except leather footwear)
Industrial products	Sheet glass and safety glass
Industrial products	Glass fiber and glass fiber products, n.e.c.
Industrial products	Miscellaneous glass products
Industrial products	Cement
Industrial products	Ready mixed concrete
Industrial products	Cement products
Industrial products	Pottery, china and earthenware
Industrial products	Clay refractories
Industrial products	Miscellaneous structural clay products
Industrial products	Carbon and graphite products
Industrial products	Abrasive and its products
Industrial products	Miscellaneous ceramic, stone and clay products
Industrial products	Pig iron
Industrial products	Ferro-alloys
Industrial products	Crude steel (converters)
Industrial products	Crude steel (electric furnaces)
Industrial products	Scrap iron
Industrial products	Hot rolled steel
Industrial products	Steel pipes and tubes

Industrial products	Cold-finished steel
Industrial products	Coated steel
Industrial products	Cast and forged steel
Industrial products	Cast iron pipes and tubes
Industrial products	Cast and forged materials (iron)
Industrial products	Iron and steel shearing and slitting
Industrial products	Miscellaneous iron or steel products
Industrial products	Copper
Industrial products	Lead and zinc (including regenerated lead)
Industrial products	Aluminum (including regenerated aluminum)
Industrial products	Miscellaneous non-ferrous metals
Industrial products	Non-ferrous metal scrap
Industrial products	Electric wires and cables
Industrial products	Optical fiber cables
Industrial products	Rolled and drawn copper and copper alloys
Industrial products	Rolled and drawn aluminum
Industrial products	Non-ferrous metal castings and forgings
Industrial products	Nuclear fuels
Industrial products	Miscellaneous non-ferrous metal products
Industrial products	Fabricated construction-use metal products
Industrial products	Fabricated architectural metal products
Industrial products	Gas and oil appliances, heating and cooking apparatus
Industrial products	Bolts, nuts, rivets and springs
Industrial products	Metal containers, fabricated plate and sheet metal
Industrial products	Plumbing accessories, powder metallurgy products and tools
Industrial products	Miscellaneous metal products
Industrial products	Boilers
Industrial products	Turbines
Industrial products	Engines
Industrial products	Pumps and compressors
Industrial products	Conveyors
Industrial products	Refrigerators and air conditioning apparatus
Industrial products	Bearings
Industrial products	Miscellaneous general-purpose machinery
Industrial products	Machinery for agricultural use
Industrial products	Machinery and equipment for construction and mining
Industrial products	Textile machinery
Industrial products	Daily lives industry machinery
Industrial products	Chemical machinery
Industrial products	Casting equipment and plastic processing machinery
Industrial products	Metal machine tools
Industrial products	Metal processing machinery

Industrial products	Machinists' precision tools
Industrial products	Semiconductor making equipment
Industrial products	Metal molds
Industrial products	Vacuum equipment and vacuum component
Industrial products	Robots
Industrial products	Miscellaneous production machinery
Industrial products	Copy machine
Industrial products	Miscellaneous office machines
Industrial products	Service industry and amusement machines
Industrial products	Measuring instruments
Industrial products	Medical instruments
Industrial products	Optical instruments and lenses
Industrial products	Ordnance
Industrial products	Semiconductor devices
Industrial products	Integrated circuits
Industrial products	Liquid crystal panel
Industrial products	Flat-panel and electron tubes
Industrial products	Storage media
Industrial products	Electric circuit
Industrial products	Miscellaneous electronic components
Industrial products	Rotating electrical equipment
Industrial products	Transformers and reactors
Industrial products	Relay switches and switchboards
Industrial products	Wiring devices and supplies
Industrial products	Electrical equipment for internal combustion engines
Industrial products	Miscellaneous electrical devices and parts
Industrial products	Household air-conditioners
Industrial products	Household electric appliances (except air-conditioners)
Industrial products	Applied electronic equipment
Industrial products	Electric measuring instruments
Industrial products	Electric bulbs
Industrial products	Electric lighting fixtures and apparatus
Industrial products	Batteries
Industrial products	Miscellaneous electrical devices and parts
Industrial products	Wired communication equipment
Industrial products	Mobile phone
Industrial products	Radio communication equipment (except mobile phones)
Industrial products	Radio and television sets
Industrial products	Miscellaneous communication equipment
Industrial products	Video equipment and digital camera
Industrial products	Electric audio equipment
Industrial products	Personal Computers

Industrial products	Electronic computing equipment (except personal computers)
Industrial products	Electronic computing equipment (accessory equipment)
Industrial products	Toys and games
Industrial products	Sporting and athletic goods
Industrial products	Jewelry and adornments
Industrial products	Watches and clocks
Industrial products	Musical instruments
Industrial products	Stationery
Industrial products	"Tatami" (straw matting) and straw products
Industrial products	Audio and video records, other information recording media
Industrial products	Miscellaneous manufacturing products
Industrial products	Office supplies
Industrial products	Activities not elsewhere classified
Other services	Veterinary service
Other services	Agricultural services (except veterinary service)
Other services	Reuse and recycling
Other services	Non-residential construction (wooden)
Other services	Non-residential construction (non-wooden)
Other services	Telecommunication facilities construction
Other services	Wholesale trade
Other services	Retail trade
Other services	Financial service
Other services	Life insurance
Other services	Non-life insurance
Other services	Real estate agencies and managers
Other services	Real estate rental service
Other services	Postal services and mail delivery
Other services	Fixed telecommunications
Other services	Mobile telecommunications
Other services	Services relating to telecommunications
Other services	Public broadcasting
Other services	Private broadcasting
Other services	Cable broadcasting
Other services	Information services
Other services	Internet-based services
Other services	Video picture, sound information, character information production (except newspapers and publication)
Other services	Newspapers
Other services	Publication
Other services	Public administration (central government)
Other services	Public administration (local government)
Other services	Membership-based business associations

Other services	Private non-profit institutions serving households, n.e.c.
Other services	Goods rental and leasing (except car rental)
Other services	Advertising services
Other services	Machine repair services
Other services	Judicial, financial and accounting services
Other services	Civil engineering and construction services
Other services	Worker dispatching services
Other services	Building maintenance services
Other services	Guard services
Other services	Miscellaneous business services
Other services	Hotels
Other services	Cleaning
Other services	Barber shops
Other services	Beauty shops
Other services	Public baths
Other services	Miscellaneous cleaning, barber shops, beauty shops and public baths
Other services	Movie theaters
Other services	Performances (except movie theaters), theatrical companies
Other services	Stadiums and companies of bicycle, horse, motorcar and motorboat races
Other services	Sport facility service, public gardens and amusement parks
Other services	Amusement and recreation facilities
Other services	Miscellaneous amusement and recreation services
Other services	Photographic studios
Other services	Ceremonial occasions
Other services	Supplementary tutorial schools, instruction services for arts, culture and technical skills
Other services	Miscellaneous repairs, n.e.c.
Other services	Miscellaneous personal services

References

1. Daigo, I.; Iwata, K.; Ohkata, I.; Goto, Y. Macroscopic Evidence for the Hibernating Behavior of Materials Stock. *Environ. Sci. Technol.* **2015**, *49*, 8691–8696.

2. Hashimoto, S.; Tanikawa, H.; Moriguchi, Y. Where will large amounts of materials accumulated within the economy go? - A material flow analysis of construction minerals for Japan. *Waste Manag.* **2007**, *27*, 1725–1738.

3. Xi, F.; Davis, S.J.; Ciais, P.; Crawford-Brown, D.; Guan, D.; Pade, C.; Shi, T.; Syddall, M.; Lv, J.; Ji, L.; et al. Substantial global carbon uptake by cement carbonation. *Nat. Geosci.* **2016**, *9*, 880–883.

4. Cao, Z.; Myers, R.J.; Lupton, R.C.; Duan, H.; Sacchi, R.; Zhou, N.; Miller, T.R.; Cullen, J.M.; Ge, Q.; Liu, G. The sponge effect and carbon emission mitigation potentials of the global cement cycle. *Nat. Commun.* **2020**, *11*, 1–9.

5. Guo, R.; Wang, J.; Bing, L.; Tong, D.; Ciais, P.; Davis, S.J.; Andrew, R.M.; Xi, F.; Liu, Z. Global CO2 uptake by cement from 1930 to 2019. *Earth Syst. Sci. Data* **2021**, *13*, 1791–1805.

6. Nansai, K.; Inaba, R.; Kagawa, S.; Moriguchi, Y. Identifying common features among household consumption patterns optimized to minimize specific environmental burdens. *J. Clean. Prod.* **2008**, *16*, 538–548.

7. Japan Cement Associate *Cement Handbook 2021*; Japan, 2021;

8. ZENNAMA Statistics Available online: https://www.zennama.or.jp/3-toukei/index.html (accessed on Dec 1, 2021).

9. Ministry of Economy Trade and Industry Statistical Survey of Crushed Stone and Other Materials Available online: https://www.meti.go.jp/statistics/sei/saiseki/index.html (accessed on Dec 1, 2021).

10. Ministry of Land Infrastructure Transport and Tourism Construction related statistics Available online: https://www.mlit.go.jp/statistics/details/jutaku_list.html (accessed on Dec 1, 2021).

11. Cabinet Office Measuring Infrastructure in Japan 2017 Available online: https://www5.cao.go.jp/keizai2/ioj/docs/ioj_docs_list.html.

12. Ministry of Land Infrastructure Transport and Tourism Survey on actual demand for construction materials and labor Available online: https://www.e-stat.go.jp/stat-search/files?page=1&toukei=00600040 (accessed on Dec 1, 2021).

13. Omi, Y.; Kurita, N. Simulation of Building Residual Ratio Under the Trends in Longer Life of Buildings. *J. Archit. Plan. (Transactions AIJ)* **2010**, *75*, 2459–2465.

14. Yukio Komatsu, Yasuhisa Kato, Takuro Yoshida, T.Y. Report of an Investigation of the Life Time Distribution of Japanese Houses at 1987. *J. Archit. Plan.* **1992**, *439*.

15. Cao, Z.; Shen, L.; Løvik, A.N.; Müller, D.B.; Liu, G. Elaborating the History of Our Cementing Societies: An in-Use Stock Perspective. *Environ. Sci. Technol.* **2017**, *51*, 11468–11475.

16. Matsui, K.; Hasegawa, M.; Takagi, S.; Okuoka, K.; Tanikawa, H. Future Scenario

Analysis of Material Stock and Flow of Construction Materials Toward Decarbonization. *J. JSCE* **2015**, *71*, II_309-II_317.

17. Ministry of Land Infrastructure Transport and Tourism Survey on actual conditions of construction Available online: https://www.mlit.go.jp/sogoseisaku/region/recycle/d02status/d0201/page_020101census.htm (accessed on Dec 1, 2021).

18. Ministry of the Environment Method for calculating greenhouse gas emissions and sinks Available online: https://www.env.go.jp/earth/ondanka/ghg-mrv/methodology/#main (accessed on Nov 19, 2021).

19. Kawai, K. LCCO2 of pavement. *Concr. J.* **2010**, *48*, 47–50.

20. Miller, S.A.; Horvath, A.; Monteiro, P.J.M. Impacts of booming concrete production on water resources worldwide. *Nat. Sustain.* **2018**, *1*, 69–76.

21. Japan Cement Associate Statistics Available online: https://www.jcassoc.or.jp/cement/2eng/e_02.html (accessed on Dec 1, 2021).

22. Ministry of Economy Trade and Industry Energy White Paper Available online: https://www.enecho.meti.go.jp/about/whitepaper/ (accessed on Dec 1, 2021).

23. International Energy Agency *World Energy Outlook 2020*; Paris, France, 2020;

24. Cao, Z.; Masanet, E.; Tiwari, A.; Akolawala, S. *Decarbonizing Concrete: Deep decarbonization pathways for the cement and concrete cycle in the United States, India, and China*; 2021;

25. Sakai, K. Current state of CO2 Emission in Concrete Sector and its Reduction Strategy. *Concr. J.* **2010**, *48*.

26. Higuchi, M. Fundamental Study on Environmental Factors in Assessing the Environmental Impact of Concrete. In Proceedings of the Japan Concrete Institute; 2002.

27. Taiheiyo Cement Mitigating Climate Change Available online: https://www.taiheiyo-cement.co.jp/english/csr/global_warm_fr.html (accessed on Apr 7, 2022).

28. Hashimoto, S.; Tanikawa, H.; Moriguchi, Y. Potential Waste-A Case Study of Construction Minerals. In Proceedings of the The Institute of Life Cycle Assessment, Japan; 2007.

29. Tanikawa, H.; Fishman, T.; Hashimoto, S.; Daigo, I. A framework of indicators for associating material stocks and fl ows to service provisioning : Application for Japan 1990-2015. *J. Clean. Prod.* **2021**, *285*, 125450.

30. Sogo, S. Toward Reduction and Effective Use of Residual Concrete Available online: https://doi.org/10.3151/coj.48.9_83 (accessed on Apr 1, 2022).

31. Fricko, O.; Havlik, P.; Rogelj, J.; Klimont, Z.; Gusti, M.; Johnson, N.; Kolp, P.; Strubegger, M.; Valin, H.; Amann, M.; et al. The marker quantification of the Shared Socioeconomic Pathway 2: A middle-of-the-road scenario for the 21st century. *Glob. Environ. Chang.* **2017**, *42*, 251–267.

32. ERMCO (European Ready Mixed Concrete Organization) *ERMCO Statistics 2018*;

Brussels, Belgium, 2019;

33. Hyodo, H.; Hoshino, S.; Hirano, H.; Nomura, K. A study on calculation of CO2 uptake due to carbonation during concrete life-cycle. *Cem. Sci. Concr. Technol.* **2021**, *74*, 333–340.

34. Hirao, H.; Hayashi, K.; Nomura, K.; Hyodo, H. Long-term Vision of Greenhouse Gas Emissions Reduction Toward 2050. *TAIHEIYO Cem. KENKYU HOKOKU* **2020**, *179*, 3–14.

35. Greenhouse Gas Inventory Office of Japan and Ministry of the Environment *National Greenhouse Gas Inventory Report of JAPAN 2021*; 2021;

36. International Energy Agency *Global Methane Tracker 2022*; Paris, France, 2022;

37. Miller, S.A. Supplementary cementitious materials to mitigate greenhouse gas emissions from concrete: can there be too much of a good thing? *J. Clean. Prod.* **2018**, *178*, 587–598.

38. Scrivener, K.; Martirena, F.; Bishnoi, S.; Maity, S. Calcined clay limestone cements (LC3). *Cem. Concr. Res.* **2018**, *114*, 49–56.

39. Miller, S.A.; John, V.M.; Pacca, S.A.; Horvath, A. Carbon dioxide reduction potential in the global cement industry by 2050. *Cem. Concr. Res.* **2018**, *114*, 115–124.

40. Shanks, W.; Dunant, C.F.; Drewniok, M.P.; Lupton, R.C.; Serrenho, A.; Allwood, J.M. How much cement can we do without? Lessons from cement material flows in the UK. *Resour. Conserv. Recycl.* **2019**, *141*, 441–454.

41. International Energy Agency *The Future of Trucks – Implications for Energy and the Environment*; 2017;

42. European Cement Research Academy; Cement Sustainability Initiative *Development of State of the Art-Techniques in Cement Manufacturing: Trying to Look Ahead; CSI/ECRA-Technology Papers 2017*; Duesseldorf, Geneva, 2017;

43. Gartner, E.; Sui, T. Alternative cement clinkers. *Cem. Concr. Res.* **2018**, *114*, 27–39.

44. Miller, S.A.; Myers, R.J. Environmental Impacts of Alternative Cement Binders. *Environ. Sci. Technol.* **2020**, *54*, 677–686.

45. Lim, T.; Ellis, B.R.; Skerlos, S.J. Mitigating CO2 emissions of concrete manufacturing through CO2-enabled binder reduction. *Environ. Res. Lett.* **2019**, *14*.

46. Kobayashi, Y. CCUS/Carbon Recycling (4) Treating CO2 as a resource Available online: https://www.yomiuri.co.jp/choken/kijironko/ckeconomy/20210406-OYT8T50094/ (accessed on Dec 22, 2021).

47. Ministry of Economy Trade and Industry Roadmap for Carbon Recycling Technologies Available online: https://www.meti.go.jp/english/press/2021/0726_003.html (accessed on Dec 1, 2021).

48. The Japan Iron and Steel Federation *JISF long-term vision for climate change mitigation*; Tokyo, Japan, 2019;

49. Wassermann, R.; Katz, A.; Bentur, A. Minimum cement content requirements: A must or a myth? *Mater. Struct. Constr.* **2009**, *42*, 973–982.

50. Obla, K.H.; Hong, R.; Lobo, C.L.; Kim, H. Should Minimum Cementitious Contents for

Concrete Be Specified? *Transp. Res. Rec. J. Transp. Res. Board* **2017**.

51. Watanabe, S.I.; Kamau-devers, K.; Cunningham, P.; Miller, S.A.; Kamau-devers, K.; Cunningham, P.; Miller, S.A. *Material Efficiency as a Means to Lower Environmental Impacts from Concrete*; NCST Policy Brief; 2021;

52. Tanikawa, H.; Hashimoto, S. Urban stock over time: Spatial material stock analysis using 4d-GIS. *Build. Res. Inf.* **2009**, *37*, 483–502.

53. International Energy Agency *Material efficiency in clean energy transitions*; Paris, France, 2019;

54. Grubler, A.; Wilson, C.; Bento, N.; Boza-Kiss, B.; Krey, V.; McCollum, D.L.; Rao, N.D.; Riahi, K.; Rogelj, J.; De Stercke, S.; et al. A low energy demand scenario for meeting the 1.5 °c target and sustainable development goals without negative emission technologies. *Nat. Energy* **2018**, *3*, 515–527.

55. Fishman, T.; Heeren, N.; Pauliuk, S.; Berrill, P.; Tu, Q.; Wolfram, P.; Hertwich, E. A comprehensive set of global scenarios of housing, mobility, and material efficiency for material cycles and energy systems modelling. **2020**, 1–16.

56. Zhong, X.; Hu, M.; Deetman, S.; Steubing, B.; Lin, H.X.; Hernandez, G.A.; Harpprecht, C.; Zhang, C.; Tukker, A.; Behrens, P. Global greenhouse gas emissions from residential and commercial building materials and mitigation strategies to 2060. *Nat. Commun.* **2021**, *12*, 6126.

57. Müller, D.B.; Liu, G.; Løvik, A.N.; Modaresi, R.; Pauliuk, S.; Steinhoff, F.S.; Brattebø, H. Carbon emissions of infrastructure development. *Environ. Sci. Technol.* **2013**, *47*, 11739–11746.

58. Akiko Ohnishi, Naoyuki Kawamura, Keijiro Okuoka, Feng Shi, H.T. Future Scenario Analysis of the Demand for Urban Structural Material Stock in Each Prefecture. *Environ. Eng. Res.* **2012**, *68*.

59. Pauliuk, S.; Heeren, N.; Berrill, P.; Fishman, T.; Hertwich, E.G. Global scenarios of resource and emission savings from material efficiency in residential buildings and cars. *Nat. Commun.* **2021**, *12*, 5097.

60. Milford, R.L.T. roles of energy and material efficiency in meeting steel industry C. targets; Pauliuk, S.; Allwood, J.M.; Müller, D.B. The Roles of Energy and Material Efficiency in Meeting Steel Industry CO2 Targets. *Environ. Sci. Technol.* **2013**, *47*, 3455–3462.

61. Eberhardt, L.C.M.; Birgisdóttir, H.; Birkved, M. Life cycle assessment of a Danish office building designed for disassembly. *Build. Res. Inf.* **2019**, *47*, 666–680.

62. Marinković, S.; Radonjanin, V.; Malešev, M.; Ignjatović, I. Comparative environmental assessment of natural and recycled aggregate concrete. *Waste Manag.* **2010**, *30*, 2255–2264.

63. AzariJafari, H.; Guo, F.; Gregory, J.; Kirchain, R. Carbon uptake of concrete in the US pavement network. *Resour. Conserv. Recycl.* **2021**, *167*, 105397.

64. Thiery, M.; Dangla, P.; Belin, P.; Habert, G.; Roussel, N. Carbonation kinetics of a bed of recycled concrete aggregates: A laboratory study on model materials. *Cem. Concr. Res.*

2013, *46*, 50–65.

65. Kuroda, Y.; Kikuchi, T. Uptake of Carbon Dioxide in the Demolished and Crushed Concrete. *Concr. Res. Technol.* **2009**, *20*, 15–22.

66. Allwood, J.M.; Cullen, J.M. *Sustainable materials – with both eyes open*; UIT Cambridge, Ed.; UIT Cambridge: Cambridge, 2012;

67. Cabrera Serrenho, A.; Drewniok, M.; Dunant, C.; Allwood, J.M. Testing the greenhouse gas emissions reduction potential of alternative strategies for the english housing stock. *Resour. Conserv. Recycl.* **2019**, *144*, 267–275.

68. Busch, P.; Kendall, A.; Murphy, C.W.; Miller, S.A. Literature review on policies to mitigate GHG emissions for cement and concrete. *Resour. Conserv. Recycl.* **2022**, *182*, 106278.

69. Liu, G.; Bangs, C.; Müller, D.B. Stock dynamics and emission pathways of the global aluminium cycle. *Nat. Clim. Chang.* **2013**, *3*, 178.

70. Nippon Slag Association Iron and Steel Slag Statistics Available online: https://www.slg.jp/e/statistics/index.html (accessed on Jan 13, 2022).

71. Plastic Waste Management Institute An Introduction to Plastic Recycling in Japan Available online: https://www.pwmi.or.jp/ei/index.htm (accessed on Jan 13, 2022).

72. Ministry of Internal Affairs and Communications Input-Output Tables for Japan Available online: https://www.soumu.go.jp/english/dgpp_ss/data/io/index.htm (accessed on Jan 15, 2022).
